# The Two Faces of Support for Redistribution in Colombia: Taxing the Wealthy or Assisting People in Need

**DOI:** 10.3389/fsoc.2022.773378

**Published:** 2022-04-27

**Authors:** Efraín García-Sánchez, Juan Carlos Castillo, Rosa Rodríguez-Bailón, Guillermo B. Willis

**Affiliations:** ^1^Mind, Brain and Behavior Research Center (CIMCYC-UGR), Department of Social Psychology, University of Granada, Granada, Spain; ^2^Center for Social Conflict and Cohesion Studies, Department of Sociology, Universidad de Chile, Santiago, Chile

**Keywords:** support for redistribution, perception of inequality, political attitudes, Colombia, inequality, ideology

## Abstract

Support for redistribution is crucial for reducing economic inequality. Despite people's desire for reducing extreme inequalities, they still have mixed opinions regarding how to do so. The aim of the article is to examine the underlying latent dimensions of support for redistribution and test its correlates to perceptions of and attitudes toward inequality. In two studies, we found that support for redistribution can be modeled as a latent construct depicting two different dimensions: one focused on taxing the wealthy and changing the income distribution schema, and other focused on assisting people in need and providing opportunities. We also found that the dimension related to taxing the wealthy (vs. assisting people in need) displayed higher internal reliability and correlated consistently with perceptions and attitudes toward inequality: the higher the support for taxing the wealthy, the higher the perceptions and concerns of inequality, and the lower the inequality-justifying ideologies. This research unveils distinct underlying dimensions of support for redistribution that shed light on different motivations that drive people's redistributive preferences.

## Introduction

Redistributive measures are considered as one of the most effective mechanisms for reducing economic inequality [Alvaredo et al., 2018; OECD (Organization for Economic Cooperation and Development), 2019]. Redistribution implies a set of taxes and social transfer policies through which the government distributes different kinds of resources among citizens (Luebker, 2014). From a rational choice perspective, implementing redistributive measures would be one of the most effective and intuitive responses to deal with increasing economic inequalities (Meltzer and Richard, 1981). From another perspective, the effective implementation of redistribution depends on historical events and political decisions made by democratic governments (Piketty, 2014; Atkinson, 2015). Under a democratic system, however, people will elect the politicians based on the political agendas they propose to handle societal problems. Therefore, support for redistribution becomes an important issue for tackling economic inequality and for the stability of political regimes.

But redistributive preferences do not always reflect people's concerns about inequality. Although people could desire lower levels of economic inequality (Norton and Ariely, 2011; Kiatpongsan and Norton, 2014), they may not support redistribution because they doubt that the government can or should address inequalities. For instance, people are likely to support governmental actions to reduce economic inequality, but they also display a general aversion toward raising taxes (Bartels, 2005), or they disapprove the social welfare spending (Fong, 2001). Beyond societal and individual differences, the coexistence of these seemingly contradictory attitudes toward redistribution can be the outcome of how people understand redistributive policies.

Support for redistribution implies a general desire for reducing inequality or demanding governmental intervention, but it can be extended by adding a procedural dimension about how to implement such redistributive measures. For instance, McCall and Kenworthy (2009) posited that people might demand government redistribution by implementing more transfers to people in poverty through raising taxes to the rich, while others might just desire different governmental actions aimed to increase opportunities, providing social insurance, and legislating labor regulations. Whilst people might agree that economic inequality should be reduced, they might disagree about how to do so: some would be more focused on taxing the well-off whereas others could be more focused on providing more opportunities for the worst-off.

Although taxation and social spending seem related in rational terms, the psychological processes behind each one are likely to differ. For instance, many people do not understand how the tax system works and fail to connect higher taxes with increasing the public spending that benefits the majority (Bartels, 2005). Similarly, support for redistribution may vary depending on which dimension people are focused on. As such, people may reject redistribution because they think that it is unfair to impose higher taxes on deserving well-off people and that it is immoral to give resources to the undeserving disadvantaged people (Sainz et al., 2019; Brown-Iannuzzi et al., 2021). Therefore, the understanding of redistribution based on one dimension or another, can moderate people's willingness to support redistributive measures.

Research in support for redistribution has a series of limitations in terms of measurement. Support for redistribution has been usually measured by survey items that capture the general desire for the government to reduce economic inequality (e.g., *The government should reduce the income differences between those with higher incomes and those with lower incomes*, ISSP ISSP Research Group, 2012). However, this indicator says nothing about specific actions that should be executed to achieve an effective redistributive scheme. Yet useful in applied research, using single-question measures makes the assumption that the construct is captured without measurement error, which obscures our understanding of the underlying meanings of support for redistribution (Steele and Breznau, 2019). Other indicators operationalize support for redistribution by focusing on attitudes toward the welfare state, social spending, progressive taxation, among others (e.g., van Oorschot and Meuleman, 2012; Rodriguez-Bailon et al., 2017; Scruggs and Hayes, 2017). This variety of indicators focuses on particular redistributive policies, which are not directly comparable to each other because they pay attention to different sides of redistribution (e.g., givers vs. receivers; people with higher and fewer resources).

Additionally, the vast majority of research about support for redistribution comes from Europe and the United States (e.g., Mccall and Orloff, 2017; Van Heuvelen, 2017; Breznau and Hommerich, 2019a), which overlooks some regions from the Global South. Although some international surveys account for countries from different regions of the world (i.e., World Values Survey), there is little development on explaining people's support for redistribution in non-European and non-Anglo-Saxon countries. Because of cultural, political and economic factors, researching on support for redistribution in highly unequal countries from the Global South can provide a unique perspective to extend our understanding of the motivational underpinnings of people's willingness to support redistribution.

This article aims to distinguish the underlying dimensions of support for redistribution and to test its association with people's perceptions and ideologies about economic inequality. In two studies, we used data from two independent samples in Colombia to examine whether support for redistribution is captured by different latent dimensions focusing on actions targeting both sides of the social ladder. In line with McCall and Kenworthy (2009), we argue that support for redistribution can be focused on the role of the government to redistribute resources by collecting more taxes from the wealthy (e.g., progressive taxation) or on the provision of more opportunities to people in poverty (e.g., social spending). We also tested the implications of these dimensions on different inequality-related perceptions and ideologies. Since political attitudes are embedded in broader belief systems, we argue that inequality-related perceptions and ideologies would influence support for redistribution differently according to the dimension faced: the responsibility of the government for taxing the wealthy or the provision of social insurance for people in need. In addition, this article provides empirical evidence to the scarce literature about support for redistribution in the Global South. In sum, this study contributes to bridging the gap between sociological and social psychological research about the underlying dimensions of redistributive preferences, which in turn, can lead to different understandings and attitudes toward redistribution.

### The Dimensions of Support for Redistribution

Support for redistribution has to do with the demand side of the distribution, that is, people's willingness to endorse public policies that rearrange the current—unequal—distribution of resources (McCarty and Pontusson, 2011). This construct is different from attitudes toward welfare, which focuses on people's evaluations about the overall institutions and organizations that warrant a social provision net that redistributes income, risks, and services (Mau, 2004). Instead, support for redistribution focuses on the specific procedural mechanisms through which the flux of resources is regulated by the government and distributed among the people (Kelly and Enns, 2010). Thus, support for redistribution is more specific than attitudes toward welfare systems, since it focuses on the people's favorability of the allocation of means, goods, and opportunities in a given society, rather than an overall acceptance of the political regime (i.e., the welfare state) (Roosma et al., 2013).

Support for redistribution can be conceptualized as an attitude that favors public policies aimed at reducing inequality (Steele and Breznau, 2019). But redistributive policies can point toward different social targets, even if they are related to the same goal of reducing inequality. Therefore, from a psychological perspective, the overall willingness to support redistribution can be better understood by looking at different underlying dimensions. For instance, support for redistribution can be focused on *who should redistribute* (e.g., the government, private organizations), *why they should do it* (e.g., beliefs, moral principles), *how to do it* (e.g., taxation, social spending), under *what conditions* (e.g., lack of opportunities, overall affluence), and who the beneficiaries are (e.g., all citizens, the disadvantaged) (Roosma et al., 2013). The particular content of redistributive policies is related to the procedures to implement redistribution (i.e., how to redistribute), which can include people's approval for taxation schemes, social spending programs, labor market regulations, and support for political regimes (Steele and Breznau, 2019). As such, support for redistribution could adopt several forms based on which specific policy or measure people are looking at.

In this vein, research suggests that support for redistribution can be understood through different latent dimensions. For instance, survey data from Japan showed that support for redistribution can be grouped into two kinds of latent profiles: one group endorsed redistribution motivated by economic self-interest, and another group motivated by ideologies (Sudo, 2020). Similarly, Jordan (2018) differentiates between people's belief in the responsibility of the government to reduce inequality—the overall desire for the government to intervene—and preferences for social spending programs—the need to address specific social policy areas (e.g., unemployment, health care). Likewise, McCall and Kenworthy (2009) posit that people's support for redistribution can be expressed as the endorsement of public policies aimed to redistribute economic resources (e.g., increase transfers to the poor or taxes on the wealthy), or as the government actions that provide more opportunities (e.g., access to services, social insurance). As such, support for redistribution can take various forms depending on the elements on which people are focused.

The underlying dimensions of support for redistribution can also be appreciated by the differences in people's attitudes toward particular redistributive measures. For instance, international survey data from 40 countries showed that people were more supportive of the role of the government to reduce inequality than to support progressive taxation (García-Sánchez et al., 2020). In Europe, people have a high consensus on supporting the role of the government in assuring employment benefits, but they have divided opinions on how to warrant pension schemes (Reeskens and van Oorschot, 2013). Indeed, despite the extreme inequalities in the United States, people are mostly unsupportive of redistribution in terms of raising taxes in a progressive taxation system (Bartels, 2005). Mccaffery and Baron (2005) argue that people are likely to engage in a kind of “no-more-taxes heuristic” because they do not fully understand the dynamics of the tax systems.

Additionally, support for redistribution can be affected by what people understand about it. As such, people in the United States became less supportive of redistribution when they attributed African American physical features and negative stereotypes to the recipients of welfare programs (Brown-Iannuzzi et al., 2017). On the contrary, people can increase their support for redistribution when they engage in deliberations that raise awareness of the benefits of such policies for all in society and when they realize the collective responsibility to contribute to the social good (Zimmermann et al., 2018). These findings suggest that support for redistribution varies largely depending on specific components on which people are focusing (e.g., beneficiaries, contributors, policies, etc.).

Although support for redistribution can focus on different aspects mentioned above, the responsibility of the government to deal with public affairs is a core issue. In this regard, survey data from one of the largest and more comprehensive studies about the role of the government —including 48 countries between 1986 and 2017— revealed that social insurance items (i.e., health-pension) and social protection items (i.e., unemployment aids, regulating prices) shared a common source of variance not accounted for the overall attitude toward the government (Breznau, 2019). This finding suggests that people perceive the government's responsibility on two dimensions: one related to regulations and another focused on assistance, as theorized by McCall and Kenworthy (2009).

Dimensions of support for redistribution could have contrasting relationships with different socio-psychological mechanisms. For instance, support for progressive taxation might be guided by self-interest (e.g., the well-off oppose it), whereas support for social security spending might be related to ideological beliefs of fairness (Alesina and La Ferrara, 2005). Similarly, support for redistribution can be undermined by poverty and wealth attributions (Kluegel and Smith, 1986), such that people reject redistributive measures because they held prejudice toward the low SES groups (Sainz et al., 2018) and Afro-American people (Brown-Iannuzzi et al., 2017). In addition, support for redistribution is also negatively related to political ideology (e.g., conservatism) because of people's affinities to the political regime (Jaeger, 2008) or because of individual differences in their motivation to justify the status quo (Jost, 2017). As such, identifying the dimensions of support for redistribution can help to gain a better understanding of the complexity of peoples' redistributive preferences.

### Measures of Support for Redistribution

Measuring support for redistribution in social sciences has traditionally relied on survey research. Given limited space in surveys, support for redistribution is usually measured through single-item indicators that reflects people's belief that the government should take responsibility for reducing inequality (e.g., “It is the responsibility of the government to reduce the differences in income between people with high incomes and those with low incomes,” see ISSP Research Group, 2012). For that reason, this question has become a standard indicator for researching attitudes toward redistribution (e.g., Kenworthy and Mccall, 2008; Luebker, 2014; Van Heuvelen, 2017; Breznau and Hommerich, 2019b).

There are other indicators in the literature used for studying support for redistribution. For instance, some indicators focus on the role of the government to take actions against inequality (e.g., “The government should take measures to reduce differences in income levels,” Dimick et al., 2018); the need to impose a progressive taxation system (e.g., “The government should increase taxes to give more help to the poor,” McCall et al., 2017; “High-income earners should pay more taxes than low income earners,” Rodriguez-Bailon et al., 2017); the general acceptance about the current redistributive structure in a given context [e.g., “we need larger income differences as incentives for individual effort” vs. “Incomes should be made more equal” (Wulfgramm and Starke, 2016); “Differences in income in (this country) are too large.” Dallinger, 2010]; or the support for specific social security policies [e.g., “Do you support more policies to increase the opportunities for children born in poor families and to foster more equality of opportunity, such as education policies?” (Alesina et al., 2018); “Should social benefits be cut in the future, should things stay as they are, or should social benefits be extended?” (Haack and Sieweke, 2018); or even by using left-right political ideology (Iglesias et al., 2013)] (see Table S1 for a non-exhaustive review of indicators about support for redistribution).

This wide variety of indicators, however, makes comparison across studies a challenging enterprise. Although different indicators aim to capture support for redistribution, they mix different dimensions about how people understand redistribution. Therefore, what is true for an indicator that focuses on progressive taxation, might differ for other indicators focused on social spending. As such, the identification of an underlying latent structure of this attitude can help to improve the research on this topic.

Measuring support for redistribution as an overall endorsement of the government's responsibility to reduce income differences has some limitations. First, it focuses on the agent of redistribution (i.e., who is responsible to redistribute), rather than on the procedures to redistribute or the target of the policies. Second, the overall desire for government responsibility can be overlapped with other related, but different, constructs (e.g., support for democracy, political trust), or can conflate people's knowledge and expectations about the role of the government (Breznau and Hommerich, 2019b). Third, from a methodological perspective, single-items add uncontrolled measurement error to the survey (Steele and Breznau, 2019), and limit the possibility of assessing measurement equivalence between several samples (Stegmueller, 2011). A latent variable approach based on several indicators can help to overcome some of these limitations. For instance, latent variables allow specifying specific procedural dimensions that state what redistribution is, which helps to avoid potential ambiguities in how people understand it. Furthermore, using latent variables allows modeling the measurement error of the scale, at the same time that provides information for testing measurement invariance across samples.

### Overview of the Current Research

This paper aims to examine the dimensionality of the support for redistribution and test its correlates with other constructs related to perception and attitudes toward inequality. Although the literature suggests that support for redistribution involves different components, we argue that the latent structure of attitudes toward redistribution involves at least two dimensions tackling differentiated social psychological processes, such as social comparison and stereotypes toward people with higher and lower socioeconomic backgrounds. As suggested by McCall and Kenworthy (2009), people might have different responses toward inequality, either by increasing the role of the government to reduce inequality; or by increasing other government actions oriented to provide for people in need. Similarly, Breznau (2019) found a consistent underlying dimension of attitudes toward governments' responsibility by controlling for social protection and social insurance. Accordingly, we posit that support for redistribution will be represented by two latent dimensions: attitudes toward the government responsibility to regulate the economy by taxing the wealthy, and attitudes toward the government obligation to assist people in need.

Through two studies, we examined the underlying dimensions of support for redistribution and tested its predictive validity on other inequality-related attitudes. In Study 1, we did a non-exhaustive review of indicators that measure people's support for redistribution and compiled the items most commonly used in the literature (see Table S1). We selected 10 items and conducted an exploratory factor analysis to identify the underlying factor structure of those items. In Study 2, we conducted a confirmatory factor analysis with an independent sample to replicate the two-dimension structure of support for redistribution. In addition, we test the reliability of the measure and the association between support for redistribution (and its dimensions) with inequality-related attitudes. This procedure is commonly used in behavioral sciences for testing psychometric properties of reliability and validity in scale validation (Bandalos, 2018). Analyses were supported by R software (R Core Team, 2020). The data, code and materials linked to this paper are available at: https://osf.io/2z98y/.

Additionally, it is worth noting that these studies were conducted in Colombia, a highly unequal Latin American country. Latin American contexts present a complex scenario for studying people's support for redistribution due to the institutional configuration of their political regimes. Indeed, given the recent experiences of authoritarian regimes and the longstanding inequalities in the Latin American region, people show low levels of political trust and are skeptical about the government's role in tackling social issues (Zmerli and Castillo, 2015; Mattes and Moreno, 2018). In Colombia, particularly, the political regime has been mainly conservative throughout the twentieth century and has experienced an armed conflict for several decades (Kajsiu, 2019), which has minimized the public discussion about redistribution from the political agenda despite being a foundational topic of the armed conflict. Between April and July 2021, however, the social unrest in Colombia increased significantly leading to large political mobilizations against regressive tax reforms, cut of social spending, and privatization of social services (Rincón, 2021).

Furthermore, as far as we know, there is little empirical research on support for redistribution in Colombia and Latin America, and the existing research did not evaluate its dimensionality. Some previous studies, for instance, have shown that people in Colombia mostly agree that the government should reduce inequality, but that such an attitude was weaker when people perceive less economic inequality and endorse system-justifying ideologies (García-Sánchez et al., 2018). However, we did not find evidence so far about people's evaluations of particular redistributive policies being discussed currently in Colombia such as the agrarian reform, the land redistribution, the educational quotas, for which there are larger levels of political polarization (Movilizatorio, 2021). As such, people might have a general agreement that the government should reduce inequality and, at the same time, have large disagreements on the specific strategies to do so.

## Study 1

The aim of Study 1 is to examine the underlying dimensions of support for redistribution based on indicators commonly used in the literature. After a non-systematic review of survey indicators that measure support for redistribution, we empirically tested how they were associated to form a latent dimension that reflects substantive attitudinal components.

### Materials and Methods

#### Participants

We used a convenience sample of 818 participants from the community of a public university in Colombia (students, academic, administrative, and maintenance staff) responded to an open call to participate in a study about current social issues in Colombia (*M*_*age*_ = 29.77, *SD*_*age*_ = 12.77, 54.58% female). Participants were contacted through the University communication office by sending them an email with an open invitation to participate in this study. The invitation said that everyone could participate voluntarily and that this study was being conducted for academic purposes, taking special care to assure anonymity and confidentiality of responses. If agreed, participants were redirected to a questionnaire uploaded in an online platform (i.e., Qualtrics), and signed informed consent before participating in the study. Although this is not a representative sample, this sampling strategy is still useful for our purposes because we aim to explore the psychometric properties of the items, rather than to draw conclusions for the whole population about their levels of support for redistribution. Data were collected during April 2018.

#### Measures

*Support for Redistribution*: Participants completed a ten-item measure of support for redistribution (M = 5.21, SD = 0.85, α = 0.737). The items covered survey indicators commonly used in the literature to operationalize support for redistribution (i.e., “The government should reduce income differences between the rich and the poor”), as well as related research that used different proxy indicators (e.g., welfare attitudes, preferences for progressive taxation and social security spending). Respondents were asked to rate their level of agreement with each statement on a 7-point scale ranging from 1 “strongly disagree” to 7 “strongly agree.” The specific wording of the items is shown in Table 1.

**Table 1:** Descriptive statistics and factor loadings of the exploratory factor analysis for the scale of support for redistribution (Study 1).

	**Item**	**Mean**	**SD**	**Skew**	**Factor 1 loadings**	**Factor 2 loadings**	**Com**.
1	The government has a responsibility to reduce the income gap between those who have more and those who have less.	5.44	1.62	−1.02	0.67		0.47
2	The Government should provide a decent standard of living for people who are unemployed.	5.31	1.46	−0.86	0.36	0.35	0.25
3	The government should spend more money on subsidies for the poor.	4.06	1.8	−0.12		0.64	0.41
4	The government should impose higher taxes on people with the highest income.	5.44	1.67	−1.05	0.38		0.21
5	Places in universities should be reserved for the most disadvantaged people.	5.42	1.61	−1.01		0.38	0.18
6	There is a great need to redistribute wealth from those who have more to those who have less.	5.19	1.71	−0.85	0.69		0.55
7	There is no need to change the distribution of economic income in Colombia (recoded)	6.3	1.13	−2.12	0.49		0.24
8	The government should increase taxes to give more aid to those most in need.	3.28	1.79	0.38		0.36	0.14
9	The richest people should help the most needy people more.	5.49	1.5	−1.01		0.46	0.29
10	The Government should do everything possible to improve the economic conditions of the most disadvantaged groups.	6.17	1.13	−1.79	0.45	0.32	0.30

*SD, standard deviation; Com., communality*.

#### Data Analysis

We conducted descriptive analyses of the items and tested the reliability of the measures. To examine the dimensionality of the scale, we conducted an exploratory factor analysis using principal-axis factor extraction with Minimum Residual procedure and varimax rotation, supported by the *psych* package (Revelle, 2018) implemented for the R software (R Core Team, 2020).

### Results and Discussion

As shown in Table 1, most of the items' mean scores are over the technical middle point of the scale (4), and are negatively skewed, which indicates that participants scored mostly on the right (“agreement”) side of the scale. However, participants tended to disagree with item 8 (“The government should increase taxes to provide more assistance to the most needed people”).

The parallel analyses, along with the visual inspection of the scree-plot and the eigenvalues over Kaiser's criterion of 1, suggest a two-factor solution for the exploratory factor analysis. Factor 1 represents the idea that the government should redistribute resources by increasing taxes (e.g., “Government should impose higher taxes on higher-income earners.”); and Factor 2 highlights the idea that the government should increase social spending and provide more assistance to people in need (e.g., “The government should spend more money on subsidies for the poor”).

We excluded items 2 and 10 from the scale because they loaded simultaneously on the two factors. Indeed, the content of those items focuses on the general idea that the government should intervene directly to ease the hardships of the disadvantaged, whereas the other items are focused on particular actions for the government toward both the wealthy and the poor. We found that removing these items from the scale provided acceptable fit indices of the model, $\chi_{\left(13\right)}^{2}$ = 56.72, *p* < 0.001, *RMSEA* = 0.054; *TLI* = 0.929.

In this regard, we found that one factor was associated with the role of the government to reduce economic inequality by redistributing resources from the wealthy toward people in poverty and by altering the overall scheme of the income distribution. The second factor grouped items related to the government's responsibility for assisting people in need, providing financial aid and opportunities. As such, the two factors of support for redistribution capture different frames of support for redistribution. On the one hand, people demand a stronger role for the government to regulate the economy by taxing the wealthy, and by providing opportunities to the needy to get ahead in life.

As for the reliability analyses, removing items did not improve the Cronbachs' alpha of the general scale (α = 0.69), which suggests that all the items contribute to the scale reliability. Concerning the components' reliability, Factor 1 related to government intervention increasing taxes of the wealthy obtained higher internal consistency (α = 0.67) than Factor 2, related to the government intervention to assist the disadvantaged (α = 0.53). This reliability analysis suggests that people were more consistent about redistribution seen as the government regulations relating to the wealthy than on the government responsibility toward people in poverty.

In addition, a paired t-test for comparing the observed means of the two factors showed that people scored higher in Factor 1 related to taxing the wealthy (*M* = 5.59, *SD* = 1.10) compared to Factor 2 about assisting people in poverty (*M* = 4.56, *SD* = 1.08), *t*_(821)_ = 24.07, 95*% CI* [−1.11, −0.094], *p* < 0.001, *d*_*Cohen*_ = −0.840^1^. Although both factors are intrinsically correlated and have implications to effectively implement redistributive policies, these findings suggest that people have different levels of endorsement for redistribution depending on which redistributive strategy they focus on.

In sum, the findings of Study 1 suggest the existence of two underlying dimensions of support for redistribution, one focused on the government's responsibility for taxing the wealthy and the other for assisting the needy. Those dimensions, however, displayed differences. On the one hand, the reliability was higher for the dimension related to taxes compared to the one related to assisting the needy. That is, the factor about taxes (vs. assistance) showed a greater degree of consistency in the relationship between items, which indicates that the items are highly interconnected and tend to reflect the same construct (Tavakol and Dennick, 2011). In addition, people were more supportive of increasing the taxation system and changing the redistribution scheme than giving more assistance to the needy. Those dimensions seem to be related to the same construct but suggest conceptual and empirical nuances.

This study has some limitations that we aim to overcome in Study 2. First, because of the nature of our research, our findings are exploratory and descriptive. Thus, we need to provide more evidence about the dimensionality and validity of the measure built for this study of support for redistribution. Second, participants in Study 1 belonged to the same public institution, which in the Colombian context, is an institution actively engaged in defending social justice issues. As such, people in this context may be informed about the role of elites in the distribution of resources, which in turn makes them more focused on taxing the wealthy than providing more assistance to people in poverty. This limitation related to the characteristics of our sample prevents us from drawing conclusions for the Colombian population. However, the contribution of this exploratory study is to get preliminary evidence about the psychometric properties of the items and their factorial structure, rather than mapping out the population attitudes about redistribution. Because using convenience samples for testing scales provide similar psychometric properties than those found in representative samples (Winton and Sabol, 2021), these findings are still useful for identifying the latent dimensions of support for redistribution. In Study 2, we try to overcome some of the shortcomings of Study 1 by testing the properties of the scale in a different, larger, and more diverse sample from Colombia. We also examined the association between both components of support for redistribution to inequality-related perceptions and attitudes.

## Study 2

The aim of Study 2 was to confirm the two-factor structure of the support for redistribution measurement in a different and more diverse sample from a broader project about perceptions of inequality and redistribution in Colombia. Regarding the predictive validity of the scale, we examined the relationship between support for redistribution and variables related to the justification of inequality, such as perceptions and ideologies about inequality, and socioeconomic status. We expected that support for redistribution would be positively associated with the perceived income gap (H_1a_), frequency of perceived inequality in daily life (H_1b_), and the general concern about inequality (H_1c_), but negatively associated with the ideal income gap (H_1d_). As for ideological variables, we expect that the support for redistribution is negatively associated with meritocracy (H_2a_), economic system justification (H_2b_), and political conservatism (H_2c_). Finally, following the self-interest hypothesis, it would be expected that support for redistribution is negatively associated with objective socioeconomic status by income (H_3a_), education (H_3b_), and subjective socioeconomic status (H_3c_). We also explored whether the strength of those associations was different depending on each dimension of support for redistribution.

### Materials and Methods

#### Participants

Participants were contacted through different universities in Colombia (19 universities, both public and private) from different cities representing the five geographical regions of the country (i.e., Bogotá D.C. Andean, Caribbean, South, Pacific). Although 2372 participants took the survey, 1901 completed the questionnaire (*M*_*age*_ = 22.02 years, *SD* = 5.53, 66.24% Female). The participants were composed both of students and workers from different levels in each university. Concerning the participants' educational profile, 81.57% were undergraduates, 11.19% were enrolled in work training, 3.75% graduated from high school, and 3.47% had postgraduate education. Participants responded to an open call delivered by each university to participate in a study about social issues in Colombia. They were informed about the goals and conditions of the research and, if agreed in taking the study, signed an informed consent and accessed the questionnaire. Considering the household income of the participants and the criteria commonly used to define social classes in Colombia (Departamento Administrativo Nacional de Estadística, 2019), we observed that 16.04% of our sample faced economic vulnerability or poverty (household below $781.242 COP), 76.7% ranged from low-middle to upper-middle (household between $1.561.000 $6.241.000 COP) and 7.26% pertained to high class (household above $6.241.000 COP). Although this sample is unrepresentative of the Colombian population, the sociodemographic characteristics offer gender, regional, and socioeconomic diversity. Data were collected between April and June 2018.

#### Measures

*Support for redistribution:* Participants responded to eight items of support for redistribution selected from study 1. Similarly to Study 1, respondents rated their level of agreement with some statements, for which they used a scale that ranged from 1 “strongly disagree” to 7 “strongly agree”.

*Economic system justification:* We used the short scale translated into Spanish by Jaume et al. (2012), which is composed of seven items regarding the legitimacy of economic inequalities in society. Participants rated each statement on a scale ranging from 1 “strongly disagree” to 7 “strongly agree” (α = 0.706). Some examples of items are: “the gap between social classes reflects the natural state of affairs of society” and “the economic position of people is a by-product of their achievements” (see Supplementary Material for the wording of all the items).

*Meritocracy*: We translated and adapted into Spanish a six-item scale of meritocracy used by Zimmerman and Reyna (2013) (α = 0.807). Participants were asked to indicate their support for several statements related to how people can get ahead in life through merit and hard work. Responses were rated according to a seven-point scale from 1 “strongly disagree” to 7 “strongly agree”. Some examples of items are: “Hard-working people achieve success in their life” and “In the Colombian society, getting ahead in life is possible for all the people that try hard”.

*Political self-positioning*: We used the left-right self-positioning scale, in which people placed themselves on a scale from 1 “Extremely left” to 7 “Extremely Right”.

*Perceptions of inequality*: We used several and different indicators of perceptions of inequality. First, we used the *perceived income gap*, which was operationalized as the ratio between the salary that participants think that earn a high status (a CEO) and a low-status (blue-collar) worker in a large company. This ratio was log-transformed to take care of some of the metric properties of this construct, as suggested by the social justice literature (Jasso et al., 2016).

Secondly, we included the *ideal income gap measure*, which was computed in the same way as the perceived income gap, but instead of asking for the perceived current earnings, they indicated how much a high-status and a low-status worker should earn.

Third, we included the *general concern of economic inequality*, which is the average of two items about people's evaluation of the extent of economic inequality (“In general, economic income differences in Colombia are too large” and “Economic income differences that I see around me are too large”), *r*_(1,954)_ = 0.721, *p* < 0.001. Respondents have to use a 7-point scale from 1 “strongly disagree” to 7 “strongly agree”.

Fourth, we used a single item to evaluate the *frequency* with which people perceive economic inequality in their daily life (i.e., “How frequent do you see situations concerning economic inequality in your daily life”) (García-Castro et al., 2019). This item was ranked on a 7-point scale from 1 “Never” to 7 “All the time.”

*Socioeconomic Status by income*: Participants indicated their approximate household income on a 10-point scale from 1 “Up to 781.242 pesos” (the minimum legal wage in 2018 in Colombia) to 10 “More than 7.021.000 pesos,” which represent a range from 261 USD to more than 2352 USD, approximately. Every point of the scale was increasing progressively from one Colombian minimum wage up to 10 times this quantity.

*Socioeconomic Status by education:* Participants indicated their educational attainment by using a 7 point scale: 1 “primary school,” 2 “High School,” 3 “work training,” 4 “undergraduates,” 5 “Specialization courses,” 6 “Master,” and 7 “Doctorate.”

*Subjective socioeconomic status:* We used the McCarthur Scale to measure subjective socioeconomic status. For this indicator, people ranked themselves in a 10-point ladder, where the option at the bottom (1) represented the group of people with the lowest salaries, educational level, and occupational prestige of the society; whereas the option at the top (10) represented the group of people with the highest salaries, educational level, and occupational prestige of the society.

#### Data Analysis

We conducted a confirmatory factor analysis with Maximum Likelihood (ML) estimator to replicate the two-factor structure of the support for redistribution measure. In addition, we tested the convergent and divergent validity of the scale by examining the relationship of each dimension of support for redistribution with other constructs related to perceptions and ideologies of inequality, and socioeconomic status. We also compared the correlation coefficients for all the variables included in the study with support for redistribution dimension (Diedenhofen and Musch, 2015).

### Results and Discussion

#### Dimensionality and Reliability of Support for Redistribution

**Figure 1:**
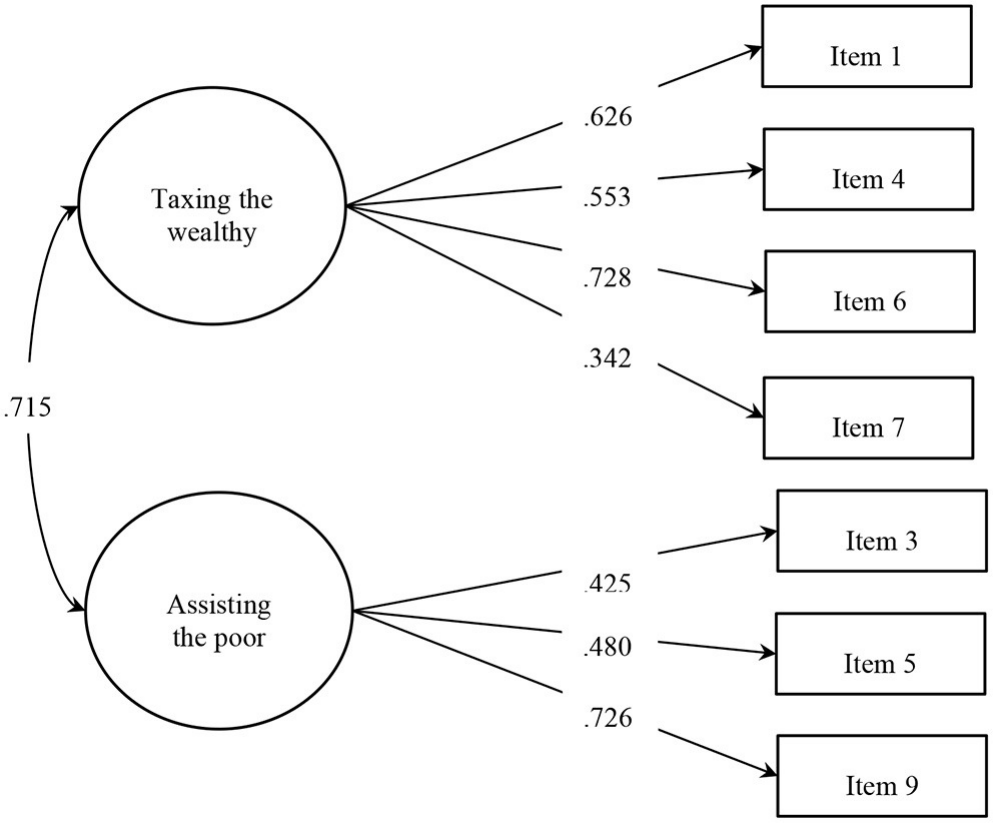
Dimensions of support for redistribution (standardized factor loadings).

We conducted a confirmatory factor analysis to estimate the two-factor model of support for redistribution: the role of the government to redistribute resources by implementing progressive taxation; and the role of the government to provide more assistance to the disadvantaged. The two-factor model obtained poor fit indices, $\chi_{\left(19\right)}^{2}$ = 248.89, *p* < 0.001, *RMSEA* = 0.080, 90*% CI* = [0.071, 0.089], *CFI* = 0.899, *TLI* = 0.851, *SRMR* = 0.050^2^. We inspected the modification indices for this model and found that the source of misfit came from item 8 (i.e., “The government should increase the taxes to provide more aids to the more disadvantaged”). Indeed, the empirical performance of this item was different from the others, since the average score was below the middle point of the scale (4) and positively skewed (*M*__*item*_8_ = 3.32, *SD* = 1.82, *Skewness* = 0.34), whereas all the other items scored above the middle point and were negatively skewed (the descriptive statistics for all the items are in Table S2). Therefore, we re-specified a two-factor model discarding item 8, which provided appropriate fit indices, $\chi_{\left(13\right)}^{2}$ = 58.61, *p* < 0.001, *RMSEA* = 0.043, 90*% CI* = [0.032, 0.055], *CFI* = 0.977, *TLI* = 0.963, *SRMR* = 0.028 (Factor loadings of Study 2 can be seen in Figure 1).

The reliability for the general scale indicates that the items were consistently associated between them (α_*Chronbach*_ = 0.698; ω_*MacDonald*_ = 0.705*; r*_*meaninter*−*item*_ = 0.251). As for the reliability of the dimensions, we found that the dimension related to government interventions for changing the distribution of incomes and taxing the rich had higher internal consistency (α_*Chronbach*_ = 0.647; ω_*MacDonald*_ = 0.662*, r*_*meaninter*−*item*_ = 0.312) than the dimension related to providing opportunities and assisting people in need (α_*Chronbach*_ = 0.546; ω_*MacDonald*_ = 0.542*; r*_*meaninter*−*item*_ = 0.293) (Items' descriptive statistics and correlations are available at Table S3). As in Study 1, we also found that people scored higher to support for redistribution by demanding the government to change the income distribution and implement progressive taxation (*M* = 5.33, *SD* = 1.13), than promoting more opportunities and assistance to people in poverty (*M* = 5.09, *SD* = 1.06), *t*_(1886)_ = 8.727, *p* < 0.001, *d* = 0.201^3^.

#### Convergent and Divergent Validity of Support for Redistribution

For presenting convergent and divergent validity for the support for redistribution measure, we computed Pearson zero-order correlations with variables related to socioeconomic status, perceptions, and ideologies of inequality. We first examined the correlations for the general scale, and then the differences between correlations between dimensions. For the 7-item general scale, we confirmed our hypotheses that support for redistribution was positively associated with perceptions of economic inequality, such as the perceived income gap (H_1a_, *r* = 0.059), the frequency perception of inequality in daily life (H_1b_, *r* = 0.178), and the general concern about inequality (H_1c_, *r* = 0.165); but negatively related to people's ideal levels of income inequality (H_1d_, *r* = −0.106). Furthermore, support for redistribution was negatively associated with ideologies of inequality, such as meritocracy (H_2a_, *r* = −0.059), economic system justification (H_2b_, *r* = −0.095), and political ideology —conservatism— (H_2c_, *r* = −0.224). We also confirmed that support for redistribution was negatively related to socioeconomic status by income (H_3a_, *r* = −0.195) and subjective socioeconomic status (H_3c_, *r* = −0.189); but it was not related to education levels (see Table 2). This pattern of correlations was virtually replicated using support for redistribution as a latent variable, except for the correlations with meritocracy and economic system justification, which became non-statistically significant (see Table S4).

**Table 2:** Zero-order correlations between support for redistribution and other variables included in Study 2, comparison of the correlation differences between dimension of redistribution, and descriptive statistics.

	**Support for**	**Dimensions of support**		**Test of correlation**		**Descriptive**		
	**redistribution**	**for redistribution**		**differences**		**statistics**		
	**General**	**Dimension**	**Dimension**					
	**scale**	**1: taxing**	**2: assisting**					**Range**
	**(7 items)**	**the wealthy**	**the needy**	**Z**	***p***	**M**	**SD**	**(Min.–Max.)**
Support for redistribution (general)						5.22	0.97	5.86 (1.14–7)
Support for redistribution (taxing the wealthy)	0.854^***^					5.33	1.13	6 (1–7)
Support for redistribution (assisting the poor)	0.801^***^	0.418^***^				5.09	1.17	6 (1–7)
Perceived income gap	0.054^*^	0.115^***^	−0.035	5.929	0.000	2.63	1.26	13.97 (−2.64 to 11.33)
Ideal income gap	−0.105^***^	−0.087^***^	−0.093^***^	0.236	0.813	1.43	0.97	10.78 (−5.01 to 5.77)
Perceived inequality in daily life	0.183^***^	0.157^***^	0.141^***^	0.636	0.524	5.47	1.22	5 (2–7)
General concern of inequality	0.163^***^	0.150^***^	0.125^***^	0.992	0.321	5.85	1.75	6 (1–7)
Meritocracy	−0.058^*^	−0.139^***^	0.066^**^	−8.155	0.000	4.03	1.21	6 (1–7)
Economic system justification	−0.099^***^	−0.167^***^	0.033	−7.966	0.000	4.19	1.01	6 (1–7)
Political ideology (left-right)	−0.219^***^	−0.281^***^	−0.069^**^	−8.597	0.000	3.49	1.19	6 (1–7)
SES by income	−0.189^***^	−0.221^***^	−0.091^***^	−5.204	0.000	3.46	2.44	9 (1–10)
SSS	−0.185^***^	−0.230^***^	−0.068^**^	−6.496	0.000	4.18	1.65	9 (1–10)
SES by education	−0.026	0.019	−0.056^*^	2.945	0.003	3.87	0.61	5 (2–7)
Sex (female)	−0.026	−0.032	−0.008	−0.941	0.346	0.66	0.47	1 (0–1)
Age	0.016	0.023	0.005	0.705	0.480	22.02	5.53	49 (17–66)

*Computed correlation used pearson-method with listwise-deletion. Shaded gray numbers indicate p values above 0.05 for the statistical test*.

* *p < 0.05*,

** *p < 0.01*,

*** *p < 0.001*.

#### Differences in the Correlations Between Dimensions of Support for Redistribution

We used bivariate Pearson correlations to statistically compare the strength of the associations between each support for redistribution dimension and inequality-related variables. Regarding economic inequality perceptions, we found that the perceived income gap was positively associated with support for progressive taxation (*r* = 0.115), but not with support for helping the disadvantaged (*r* = −0.035^n.s.^). The correlations coefficients between support for redistribution dimensions and perceive inequality in daily life, concern about inequality, and ideal income gap, were not different between the two support for redistribution dimensions (see Table 2).

As for ideologies about inequality, we found that meritocracy was negatively associated with taxing the wealthy (*r* = −0.139), but positively related to helping the disadvantaged (*r* = 0.066). In addition, economic system justification was negatively associated with redistribution as taxing the wealthy (*r* = −0.167), but such association became non-significant for assisting the disadvantaged (*r* = 0.033). Additionally, both dimensions of support for redistribution were negatively linked to political conservatism, but such association was stronger in relation to taxing the wealthy (*r* = −0.281) than assisting the disadvantaged (*r* = −0.069).

Socioeconomic by income and subjective socioeconomic status were also negatively correlated to both dimensions of support for redistribution, but such association was more pronounced for the dimension related to taxing the wealthy (income, *r* = −0.221; subjective, *r* = −0.230) than to the dimension of helping the disadvantaged (income, *r* = −0.091; subjective, *r* = −0.068). Finally, socioeconomic status by education was negatively associated with assisting the disadvantaged (*r* = −0.056), but not with taxing the wealthy (*r* = 0.019). These correlations were replicated by using latent variables for each support for redistribution dimension (see Table S4).

## General Discussion

In this article, we sought to test a measure of support for redistribution in two independent samples living in a highly unequal context, such as Colombia. We aimed to examine the underlying dimensions of support for redistribution, as well as to test some of its correlates with inequality-related perceptions and ideologies. Our findings confirmed that support for redistribution can be modeled as a latent variable composed of two different dimensions: taxing the wealthy and assisting the disadvantaged. We also show that there are differences between those dimensions of support for redistribution, both on the level of people's endorsement and its correlates with other inequality-related constructs. Our findings contribute to expanding the discussion about people's support for redistribution in a Latin American country, such as Colombia, which provides one of the few empirical studies on this topic in the region. These findings bring some theoretical and methodological issues to advance our understanding of support for redistribution.

First, our findings suggest that support for redistribution, as an attitude, reflects a latent construct that depicts different dimensions: one focused on government regulations and taxation, and the other focused on government responsibility to provide opportunities to people in need. These findings are aligned with previous research arguing that support for redistribution can be understood as a latent construct (Breznau, 2019; Steele and Breznau, 2019). Indeed, after controlling for crucial context-dependent items, the government's responsibility was found to reflect an invariant latent dimension across different countries and years (Breznau, 2019). Besides, observing two latent dimensions of support for redistribution suggest that people may—in general— agree on the goals (e.g., reducing inequality) but disagree about the means (e.g., particular policies aimed to reduce inequality). As such, our findings suggest that support for redistribution can be captured by different dimensions. As posited by McCall and Kenworthy (2009), support for redistribution can be directed toward changing the income distribution and implementing progressive taxes, or by giving more opportunities and financial aid to the needy.

Measuring support for redistribution can be a challenging endeavor because of the complexity of interconnected concepts embedded in redistributive schemes (e.g., actors, beneficiaries, strategies, etc.). Therefore, using single items to measure support for redistribution can be too abstract or too general for capturing people's redistributive preferences. Thus, distinguishing between the two dimensions of attitudes toward redistribution is relevant both for understanding people's redistributive attitudes and for the comparability of results from different studies. That is, from a theoretical perspective, support for redistribution is not a single attitude, but it depends on different ideas on how the world should work, along with ideological beliefs and fairness principles. From a methodological perspective, it is easy to fall into confusion based on the “face validity” of indicators: as they look similar, they should refer to the same concept (Steele and Breznau, 2019). This assumption, however, should be empirically tested to make valid statements regarding what drives people to support redistribution. We think that this discussion about the dimensionality of redistribution has been overlooked because studies in this field commonly rely on single indicators that may not fully capture the different ideas people have about redistribution. Therefore, stating what people focus on when they think about redistributive policies (i.e., dimensions) can extend our understanding of what drives people's attitudes toward redistribution. In this sense, we believe that conceptual and methodological contributions from social psychology to this area can help to bring some insights that open new research possibilities in a multidisciplinary framework.

Second, the measure of support for redistribution we present in the current research articulates the intention of reducing economic inequality with two specific ways to do so, depicted in the two latent dimensions. Yet related, these dimensions showed important nuances related to people's attitudes toward redistribution. One of the nuances is that people are more supportive of the government to change the income distribution and implement progressive taxes toward the wealthy than to support the role of the government to level up the opportunities of the disadvantaged. Previous research has shown that people display some kind of tax aversion (Fong, 2001; Bartels, 2005; Mccaffery and Baron, 2005). For instance, people from different countries are less likely to support progressive taxation schemes than to support a general claim that the government should reduce inequality (García-Sánchez et al., 2020).

In our case, however, we found a different pattern since participants were more supportive of progressive taxes than social insurance programs. This might be due to the characteristics of our sample because the participants were highly educated people who were aware of the taxes role in controlling inequality; or might be due to the group targeted for the specific public policies mentioned, which activates different stereotypes: progressive taxes are focused on the wealthy, and social insurance programs are related to the needy. That is, the differences in support for redistribution depending on the dimension people focus on can be driven by stereotypes linked to each targeted group (e.g., “undeserving rich” or “lazy low SES groups”). In this vein, international data showed that justifying the low incomes of people in poverty reduced support for redistribution more than justifying the high incomes of the wealthy, suggesting a stronger role of prejudice toward people in poverty (García-Sánchez et al., 2021). This rationale should be tested in further research to unfold particular motivations behind different dimensions of support for redistribution. As such, focusing on different dimensions of redistribution can extend our understanding of how people make sense and support different types of redistributive policies. What is more, this discussion can help us to deepen on the different social psychological mechanisms that explain not just whether people support redistribution, but which type of redistribution and to whom it should be directed.

Another nuance shown by the differences between the two dimensions revealed of support for redistribution has to do with the internal consistency and validity of each dimension. On the one hand, the dimension related to government regulations and progressive taxation displayed more internal consistency than the other related to assisting the needy. This finding can be explained by the few items that reflect each dimension, but also by the overall agreement of the people that advocates a progressive taxation scheme in a country with a poor welfare state and extreme inequalities. As such, although preliminary evidence suggest that people are likely to support redistribution in Colombia (García-Sánchez et al., 2018), when it comes to the particular ways to do it, they seem to advocate more consistently for an overall change in the income distribution than for reinforcing the public social safety net. Indeed, the low consistency for the dimension aimed at assisting the needy can be related to the diversity of opinions fed by a neoliberal ideology broadly shared in Western societies (Azevedo et al., 2019) or system-justifying beliefs, such as justification economic system (Jost, 2017) or belief in a just world (García-Sánchez et al., 2021) which blame people in poverty for their situation and downsize the importance of public spending.

A third aspect related to the two-dimensional structure of support for redistribution concerns its convergent and divergent validity. Particularly, the strength of the correlation of inequality-related attitudes with support for redistribution was higher when it was framed in terms of progressive taxes than in terms of assisting the needy. Indeed, in some cases were different since meritocracy and economic system justification beliefs were in the expected direction—negative correlation—for the case of progressive taxes, but was the opposite of what we could have expected for the case helping the disadvantaged. Such findings suggest that support for redistribution might be driven by different social psychological processes depending on the target group of the redistributive policies. On the one hand, the idea of redistribution of resources by taking money away from the rich can reflect self-interest and system justification motives: the higher the socioeconomic status and system justifying ideologies, the less support for redistribution (Brandt et al., 2020). On the other hand, social spending might be related to intergroup processes that maintain social inequalities such as social dominance, prejudice toward the disadvantaged, and attributions of poverty (Sidanius et al., 2016; Sainz et al., 2019). It is also plausible that the positive association between the assisting the disadvantaged dimension and meritocracy/economic system justification beliefs reveal a context-dependent issue. Particularly, the social mobility discourse plays an important role in the Colombian national narrative, which praises the people that “make themselves.” As such, it is possible that people that support social insurance programs also endorse such meritocratic discourse when everyone can get ahead in life by their effort. Further research could explore in-depth the particular antecedents and potential mechanisms that uniquely predict each dimension of support for redistribution.

A fourth discussion point concerns the interpretation of support for redistribution in a highly unequal context, such as Colombia. Although we expect that the two-dimension model of support for redistribution could be replicated internationally, it is also plausible that the assumptions about support for redistribution made from western, rich, democratic and industrialized societies have differences in societies with a poor state capacity and persistent economic and social exclusion patterns, such as the case of Latin American countries (Morgan and Kelly, 2017). For instance, while in Europe, support for redistribution was low in countries with less (vs. more) consolidated welfare systems (Van Heuvelen, 2017); in Latin America, support for redistribution was low in countries with more inter-ethnic inequality, with low political diversity, and with positive evaluations of the economic performance (Morgan and Kelly, 2017). Although support for redistribution are likely to be positively correlated to social spending in Anglosaxon countries (Alesina and Angeleto, 2005); in Latin America, redistributive policies are also constrained by international financial institutions that feed market inequality (Morgan and Kelly, 2013). As such, support for redistribution in Latin America is not only related to people's awareness of inequality or direct endorsement of redistributive policies, but it is also affected by a long-standing beliefs and policies that discredit social spending under neoliberal agendas that has created a political architecture that discourages the social safety net (Franzoni and Sánchez-Ancochea, 2018). Thus, support for redistribution can be an ideological measure related to political identities and symbolic components rather than to “rational” self-serving public policies preferences (Brandt et al., 2019).

This research and results have some limitations. First, the characteristics of the samples in our studies, yet social and culturally diverse, are not representative of Colombian society. Thus, it is not possible to make generalizations regarding levels of support for redistribution for the whole population. However, non-representative samples are still useful for testing the psychometric properties of a scale because these studies are focused on the measurement characteristics, rather than on the results of the scale (Winton and Sabol, 2021). That is, the structure and validity evidence of a measure rely on the consistency between indicators and its capacity to reflect expected relationships with other related constructs (Bandalos, 2018). Ideally, a measure that accurately captures a construct should display measurement invariance across samples. Therefore, we argue that our findings about the dimensionality of redistribution could be extended to other samples, although we also acknowledge that changes might be found due to social or cultural variations. However, this is an empirical question that should be addressed in further research, as it was done with similar constructs related to government responsibility (Breznau, 2019).

Another limitation has to do with the limited representativeness of items included in our measure. Although we selected a few items to examine the latent dimension of support for redistribution, those items were extracted from a large compilation of indicators used in sociology, political science, and social psychology to approach this topic (see Table S1). Therefore, it is still possible that other latent dimensions emerge in people's support for redistribution by including other items. As is the case of the multidimensionality of welfare attitudes (Roosma et al., 2013), support for redistribution could provide more dimensions related to who is responsible to redistribute (government vs. private organizations), the procedures to collect the resources (taxes vs. donations), the beneficiaries from redistribution (everyone vs. disadvantaged), and can even include moral and procedural dimensions (e.g., fairness of the redistribution, procedures efficiency, etc.). Our findings, however, seem to identity at least two dimensions that tap into two processes studied in social psychology: Intergroup processes (e.g., social class-based stigma and prejudice) and system-justifying motives (e.g., ideologies). As such, further research should take into account these distinctions to offer a more comprehensive picture of the redistributive attitudes.

Finally, it is important to acknowledge that redistributive measures are one of the most effective ways to reduce economic inequality. As such, increasing support for redistribution, under the democratic principle will lead people to support political agendas that invest in redistributive policies aimed at reducing economic inequality (Alesina and Giuliano, 2009). Therefore, our findings point out practical implications since people mostly agree on the need of reducing inequality, but disagree on the means to achieve such a goal. Increasing people's knowledge about how the redistributive system works and how it benefits society as a whole can be a promising avenue for future research and applications. For instance, despite people's reluctance to taxes, when they understood how they contribute to the well-being of the whole society, they were willing to change their minds and support more such policies (Zimmermann et al., 2018). Therefore, increasing people's awareness of inequality and understanding the mechanisms to deal with it can be a potential way to improve people's attitudes toward redistribution.

## Data Availability Statement

The datasets generated and analyzed for this study, along with code and materials, can be found at: https://osf.io/2z98y/.

## Ethics Statement

The studies involving human participants were reviewed and approved by University of Granada Ethics Committee (170/ CEIH/2016). The patients/participants provided their written informed consent to participate in this study.

## Author Contributions

EG-S arranged data collection, conducted formal analyses, and wrote the first draft of the manuscript. All authors conceived the idea of this manuscript together. All authors provided critical feedback for the manuscript and contributed to the final version equally.

## Funding

This research was supported by the Colombian COLCIENCIAS Scholarship (769) given to the first author. Complementary, this research received support from Research Grants coming from Governmental Funding Agencies in Spain (PID2019-105643GB-I00, PCI2020-112285, and B-SEJ-128-UGR18) and Chile (COES, CONICYT/FONDAP/15130009, and FONDECYT 1210847).

## Conflict of Interest

The authors declare that the research was conducted in the absence of any commercial or financial relationships that could be construed as a potential conflict of interest.

## Publisher's Note

All claims expressed in this article are solely those of the authors and do not necessarily represent those of their affiliated organizations, or those of the publisher, the editors and the reviewers. Any product that may be evaluated in this article, or claim that may be made by its manufacturer, is not guaranteed or endorsed by the publisher.

## Footnotes

^1^Given that latent means are set arbitrarily, we decided to present the differences between observed means because they are directly interpretable in the original variable scale. For robust check, however, we estimated the latent factor means and found similar results, that is: latent mean of Factor 1 related to taxing the wealthy (*M* = 5.44) was higher than the latent mean of Factor 2 related to assisting disadvantaged people (*M* = 4.06), and such difference was statistically significant, *difference* = 1.38, *SE* = 0.076, *z*= 18.12, *95% CI* = [1.23, 1.53], *p* < 0.001.

^2^$\chi_{\left(\text{df}\right)}^{2}$ = Chi square and degrees of freedom; *p* = *p* value; RMSEA = Root Mean Square Error of Approximation; CFI = Comparative Fit Index; TLI = Tucker-Lewis Index; SRMR = Standardized Root Mean Residual.

^3^As in Study 1, this difference was double-checked by using latent factor means and we found similar results: The latent factor mean for taxing the wealthy (*M* = 5.33) was higher than the factor mean for assisting the disadvantaged (*M* = 4.15), and such difference was statistically significant, *difference* = 1.177, *SE* = 0.05, *Z* = 23.68, *95% CI* = (1.08, 1.27), *p*<*0*.001.
