# Supplementary material for: The Two Faces of Support for Redistribution in Colombia: Taxing the Wealthy or Assisting People in Need
Source: Front Sociol. 2022 Apr 27;7:773378. doi: 10.3389/fsoc.2022.773378 (PMC9092524; doi:10.3389/fsoc.2022.773378)
Supplement: Supplementary file 1 [file Data_Sheet_1.PDF]

Supplementary Material

The two faces of support for redistribution in Colombia: Taxing the wealthy or assisting people in poverty

Efraín García-Sánchez[1*], Juan Carlos Castillo[2], Rosa Rodríguez-Bailón[1], Guillermo B. Willis[1]

[1] Mind, Brain and Behavior Research Center (CIMCYC-UGR), Department of Social Psychology, University of Granada, Spain

[2] Department of Sociology, Center for Social Conflict and Cohesion Studies, University of Chile, Santiago de Chile.

*** Correspondence:**
Efraín García-Sánchez
egarcias@ugr.es

Table of contents

Table S1. Indicators measuring support for redistribution (and proxys) collected from the literature 2

Table S2. Descriptive statistics for the items of support for redistribution 30

Table S3. Descriptive statistics for the items of support for redistribution and inter-item correlation in Study 2 31

Table S4. Correlations between latent variables of support for redistribution and other variables 32

Note: Supplementary Data and Material can be downloaded from: https://osf.io/2z98y/

Table S1. Indicators measuring support for redistribution (and proxys) collected from the literature

Source	Original concepetulization of the measure	Topic	Items wording
van der Toorn (2015)	Support for universal welfare (by allocating limited funds)	Social spending	the government is currently making its annual budget and has set aside a certain amount of money to be split between Human Rights Advocate groups and Road Maintenance. Last year, this money was split 50/50.'' We selected road maintenance as the alternative to human rights because it is ideologically neutral and considered generally valuable. Participants then indicated on slider scales (which added up to 100%), what percentage the government should allocate for each issue this year.
Kuhn (2015)	Attitudes to inequality	Government role	support of government intervention to reduce income diferences
Kuhn (2015)	Attitudes to inequality	Taxation	support of progressive taxation
Kteily et al. (2016)	Social Welfare Support (alpha=,77)	Social spending	Support for: "Greater assistance to the poor"
Kteily et al. (2016)	Social Welfare Support (alpha=,77)	Social spending	"Reduced public support for the homeless" (reverse-scored)
Kteily et al. (2016)	Social Welfare Support (alpha=,77)	Social spending	"Reduced benefits for the unemployed" (reverse-scored)
Kteily et al. (2016)	Support for Egalitarian Social Policy	Social spending	"Those at the top of this society should share more of their wealth with those at the bottom"
Kteily et al. (2016)	Support for Egalitarian Social Policy	Redistribution structure	"There is a need to redistribute wealth from the top to the bottom in this society"
Kteily et al. (2016)	Support for Egalitarian Social Policy	Redistribution structure	"There is no need to change the income distribution in this society." (reverse-coded)
Kteily et al. (2016)	Support for Egalitarian Social Policy	Redistribution structure	"There is a need to flatten the hierarchy in this society."
Kteily et al. (2016)	Support for Egalitarian Social Policy	Social spending	"Support should be given for those at the bottom of this society to receive more money."
Zimmerman & Reyna (2013)	support for hierarchy-enhancing policies (alpha=,59)i.e., policies intended to maintain and perpetuate inequality)	Government role	"The government should increase military spending,"
Zimmerman & Reyna (2013)	support for hierarchy-enhancing policies (alpha=,59)	Taxation	"The government should give tax breaks to large corporations,"
Zimmerman & Reyna (2013)	support for hierarchy-enhancing policies (alpha=,59)	Government role	"The government should dismantle affirmative action,"
Zimmerman & Reyna (2013)	support for hierarchy-enhancing policies (alpha=,59)	Social spending	"The government should cut spending on social programs for the poor"

Table S1. (*Continued*)

Source	Original concepetulization of the measure	Topic	Items wording
Zimmerman & Reyna (2013)	Hierarchy-Attenuating Policy Scale (alpha=.76) (i.e., policies intended to redistribute resources and reduce inequality).	Social spending	"The government should increase support for people who are unemployed,"
Zimmerman & Reyna (2013)	Hierarchy-Attenuating Policy Scale (alpha=.76)	Social spending	"The government should spend more money on pre-school or other early education programs in poor neighborhoods,"
Zimmerman & Reyna (2013)	Hierarchy-Attenuating Policy Scale (alpha=.76)	Taxation	"The government should make wealthy people pay higher taxes,"
Zimmerman & Reyna (2013)	Hierarchy-Attenuating Policy Scale (alpha=.76)	Social spending	"The government should create a free universal health care system"
Zimmerman & Reyna (2013)	Hierarchy-Attenuating Policy (Study 1) Note. R reverse scored. Adapted from General Social Surveys, 1972–2010: Cumulative Codebook (pp. 678, 2110, 2226, and 2227), by T. W. Smith, P. V. Marsden, M. Hout, and J. Kim, 2011, Chicago, IL: National Opinion Research Center. Reprinted with permission.	Social spending	1. We should not spend any more federal money on programs that assist Blacks.
Zimmerman & Reyna (2013)	Hierarchy-Attenuating Policy (Study 1)	Social spending	2. The government should increase support for people receiving food stamps.
Zimmerman & Reyna (2013)	Hierarchy-Attenuating Policy (Study 1)	Social spending	3. The government should spend more money on preschool or other early education programs in poor neighborhoods.
Zimmerman & Reyna (2013)	Hierarchy-Attenuating Policy (Study 1)	Social spending	4. The government should provide special college scholarships for children from economically disadvantaged backgrounds who maintain good grades.
Zimmerman & Reyna (2013)	Hierarchy-Attenuating Policy (Study 1)	Social spending	5. The government should provide a job to anyone who wants one.
Zimmerman & Reyna (2013)	Hierarchy-Attenuating Policy (Study 1)	Social spending	6. The government should create a free universal health care system.
Zimmerman & Reyna (2013)	Hierarchy-Attenuating Policy (Study 1)	Social spending	7. The government should not have to provide housing for those who cannot afford it. ®

Table S1. (*Continued*)

Source	Original concepetulization of the measure	Topic	Items wording
Zimmerman & Reyna (2013)	Hierarchy-Attenuating Policy (Study 1)	Social spending	8. The government should strive to hire people of color more.
Zimmerman & Reyna (2013)	Hierarchy-Attenuating Policy (Study 1)	Social spending	9. The government should provide more chances for children from poor families to go to college.
Zimmerman & Reyna (2013)	Hierarchy-Attenuating Policy (Study 1)	Social spending	10. The government should spend less on benefits for the poor. ®
Zimmerman & Reyna (2013)	Hierarchy-Attenuating Policy (Study 1)	Social spending	11. The government should provide a decent standard of living for the unemployed.
Laurin et al. (2013)	Support for redistributive programs	Social spending	Support for programs: Tutoring, Support creation; vote for government funding, Volunteer, Donate money, Soup Kitchen, Job training, Mentorship, Crisis hotline, Adopt-a-grandparent
Steele (2015)	Support for redistribution (based on ISSP)	Government role	"It is the responsibility ofthe government to reduce the differences in income between people with high incomes and those with low incomes"
Steele (2015)	Support for redistribution (based on ISSP)	Social spending	"The government should provide a decent standard ofliving for the unemployed"
Kerr (2014)	Proxy social preferences for government-led income redistribution	Government role	Responsibility of the government in the transfer of income (Government Responsibility)
Kerr (2014)	Proxy social preferences for government-led income redistribution	Taxation	progressive nature of taxation (Progressive Taxation)
Kerr (2014)	Proxy social preferences for government-led income redistribution	Inequality acceptance	the acceptability ofcurrent income differences (Inequality Acceptance)
Wulfgramm & Starke (2016)	attitudes towards redistribution	Inequality acceptance	'We need larger income differences as incentives for individual effort (1)' versus 'Incomes should be made more equal (10)'.
Gonthier (2016)	Support for redistribution	Government role	the government should take measures to reduce differences in income levels'

Table S1. (*Continued*)

Source	Original concepetulization of the measure	Topic	Items wording
Kuzyemko et al (2015)	Preferences for redistribution	Taxation	To emphasize that higher income taxes on the well-off need not always lead to slower economic growth, we presented respondents a figure showing that, at least as a raw correlation, economic growth, measured by average real pre-tax income per family from tax return data, has been slower during periods with low top tax rates (1913–1933 and 1980–2010) than with high top tax rates (1933–1980).<br><br>"Now we would like to ask you about the income tax rates that you think different peole SHOULD pay. The income tax rate is the percentage of your income thatyou pay in federeal income tax. For example, if you earn $30,000 and you pay $3,000 in income taxes, your income tax rate is 10%. Please use the sliders below to tell us how much you think each of the following groups should pay as a percentage of their total income: a top 1% (richest); The next 9% (1% of households earn more than them, 90% earn less; The next 40% (10% earn more than them, 50% earn less); The Bottom 50% (poorest)"
Schmidt-Catran (2014)	demand for redistribution	Government role	'The government should take measures to reduce differences in income levels'.
Rodriguez-Bailon et al. (2017)	Economic resource-distributionfairness	Inequality acceptance	"To what extent do you think that the resource distribution in Spanish society is just/fair?"
Rodriguez-Bailon et al. (2017)	social welfare	Social spending	"assume the government currently spends a certain amount of its budget on each of the following social welfare policies for the financially disadvantaged." They were then presented with –100 to 100 scales of six primary social welfare policies (healthcare benefits and subsidies; unemployment benefits; food stamps; social security; education grants, scholarships, and subsidized loans; public housing) and were required to indicate the amount that the government should increase (toward 100) or decrease (toward –100) spending.
Rodriguez-Bailon et al. (2017)	Government policy	Social policies	Extent to which you agree or disagree with each of the following policies:<br>class-based affirmative action (or positive discrimination) in universities, by reserving some student places for the financially or socioeconomically disadvantaged
Rodriguez-Bailon et al. (2017)	Government policy	Social policies	Class-based affrimativa action (or positive discrimination) in workplaces, by reserving some employement positions for the financially or socioeconomically disadvantaged

Table S1. (*Continued*)

Source	Original concepetulization of the measure	Topic	Items wording
Rodriguez-Bailon et al. (2017)	Government policy	Social policies	Wage subsidies, whereby the government pays employers to hire people from disadvantaged groups, to increase the number of jobs for low-skill workers
Rodriguez-Bailon et al. (2017)	Government policy	Social policies	Wage subsidies, whereby the government pays employers to train people from disadvantaged groups, to increase the number of jobs for low-skill workers
Rodriguez-Bailon et al. (2017)	Income tax	Taxation	Progressive income taxation means that the more income you earn, the more tax you pay. Please indicate the extent to wich you agree or disagree with ech of theh following statements
Rodriguez-Bailon et al. (2017)	Income tax	Taxation	High-incomee earners should pay more tax than low income earners
Rodriguez-Bailon et al. (2017)	Income tax	Taxation	The current system of progressive income tax is not fair
Rodriguez-Bailon et al. (2017)	Income tax	Taxation	Progressive income tax isi a good way to make society more equal.
Rodriguez-Bailon et al. (2017)	Income tax	Taxation	Progressive income tax is bad because it will case high-wealth individuals to move cuntries to avoid taxation taking money away from our countru
Rodriguez-Bailon et al. (2017)	Income tax	Taxation	Progressive income tax will provide more benefit to the country's economy than harm
Rodriguez-Bailon et al. (2017)	Estate tax	Taxation	Estate tax isi a good way to reduce the concentration of wealth over generations
Rodriguez-Bailon et al. (2017)	Estate tax	Taxation	Estate tax interferes with wealthy families' ability to provide for their childre
Rodriguez-Bailon et al. (2017)	Estate tax	Taxation	In 2014 the state tax exclusin amount is $5,340,000 up to which no tax burden is incurred ,and after which it incurs a 40% tax rate. This is a reasonable penalty for heirs to bear
Rodriguez-Bailon et al. (2017)	Estate tax	Taxation	Estate tax is unfair because the wealth has already been taxed
Rodriguez-Bailon et al. (2017)	Estate tax	Taxation	Estate tax is good because it promotes donations to charity to avoid taxation
Rodriguez-Bailon et al. (2017)	Luxury tax	Taxation	Luxury goods tax isi a good way to redistribute wealth

Table S1. (*Continued*)

Source	Original concepetulization of the measure	Topic	Items wording
Rodriguez-Bailon et al. (2017)	Luxury tax	Taxation	Luxury goods tax is unfair as it mostly affects the wealthy
Rodriguez-Bailon et al. (2017)	Luxury tax	Taxation	Luxury goods taxx does little to make society more equal
Rodriguez-Bailon et al. (2017)	Luxury tax	Taxation	Luxury goods tax causes more problems for society than it is worth in revenue generated
Rodriguez-Bailon et al. (2017)	Corporate taxes	Taxation	Corporate tax should be progressive, whereby compnies that earn more income should be taxed more than comanies that earns less income
Rodriguez-Bailon et al. (2017)	Corporate taxes	Taxation	Corporate tax is a good way to redistribute wealth
Rodriguez-Bailon et al. (2017)	Corporate taxes	Taxation	Taxing companies based on their level of income will discourage business growth and development
Rodriguez-Bailon et al. (2017)	Corporate taxes	Taxation	Progressive corporate ta is bad because it wil mean the more profitable companies will relocate to other countries to avoid taxation
Rodriguez-Bailon et al. (2017)	Corporate taxes	Taxation	Corporate tax will provide more benefit to the countr's economy than harm
Rodriguez-Bailon et al. (2017)	Charitable giving	Social spending	Participants had ahypothetical $100 to distribute among four charities. Two charities (United Way, Salvation Army) were considered redistributive, aiding the financially disadvantaged; the other two (American Cancer Society, Nature Conservancy) were considered nonredistributive charities. In addition, respondents indicated how “important” they thought each charity was (1 = not at all important, 7 = very important).
Guillaud (2013)	attitudes towards redistribution	Government role	“On the whole, do you think it should or should not be the government’s responsibility to reduce income differences between the rich and the poor?”
Kuhn (2012)	Support for redistribution	Government role	It is the responsibility of the government to reduce the differences in income between people with high incomes and those with low incomes.

Table S1. (*Continued*)

Source	Original concepetulization of the measure	Topic	Items wording
Kuhn (2012)	Support for progressive taxation	Taxation	Do you think people with high incomes should pay a larger share of their income in taxes than those with low incomes, the same share, or a smaller share?"
Kuhn (2009)	Support for the welfare state	Government role	It is the responsibility of the government to reduce the differences in income between people with high incomes and those with low incomes.
Bamfield & Horton (2009)	Attitudes to tackling economic inequality	Inequality acceptance	Those on high salaries do not deserve them
Bamfield & Horton (2009)	Attitudes to tackling economic inequality	Inequality acceptance	High salaries are too much because they are more than anyone else
Bamfield & Horton (2009)	Attitudes to tackling economic inequality	Taxation	Government should increase tax at the top to ensure they contribute more to public services
Bamfield & Horton (2009)	Attitudes to tackling economic inequality	Taxation	Government should use tax and benefits to reduce the income gap between rich and poor
Bamfield & Horton (2009)	Attitudes to tackling economic inequality	Government role	Government should ensure there is a maximum wage
Bamfield & Horton (2009)	Attitudes to tackling economic inequality	Social spending	Those in poverty are in genuine need
Bamfield & Horton (2009)	Attitudes to tackling economic inequality	Social spending	Benefit recipients will make a contribution back in future
Bamfield & Horton (2009)	Attitudes to tackling economic inequality	Social spending	Some people face insurmountable barriers because of their background
Bamfield & Horton (2009)	Attitudes to tackling economic inequality	Social spending	Government should increase benefits for the poorest, even if it means tax rises for everyone else
Bamfield & Horton (2009)	Attitudes to tackling economic inequality	Social spending	There should be positive action in university admissions
McCall et al. (2017)	Views on government redistribution (drawn from the core module of the GSS and the standard measure in the survey literature on views ofgovernment redistribution)	Government role	government responsibility to reduce income differences between the rich and poor on a seven-point scale (seeMethods for exactwording)

Table S1. (*Continued*)

Source	Original concepetulization of the measure	Topic	Items wording
McCall et al. (2017)	Government and Business Responsibility. Following is the EQWLTH variable in the GSS, with bracketed text denoting a substitution to generate the parallel business responsibility item	Government role	"Some people think that the government [major companies] ought to reduce the income [pay] differences between the rich and the poor [employees with high pay and those with low pay], perhaps by raising the taxes of wealthy families [reducing the pay of executives] or by giving income assistance to the poor [increasing the pay of unskilled workers]. Others think the government [major companies] should not concern itself [themselves] with reducing this income [pay] difference between the rich and the poor [employees with high pay and those with low pay]. Think of a score of 1 meaning that the government [major companies] should not concern itself [themselves] with reducing income [pay] differences, and a score of 7 as meaning that the government [major companies] ought to reduce the income [pay] differences between rich and poor [employees with high pay and those with low pay]. What score between 1 and 7 comes closest to the way you feel?"
McCall et al. (2017)	Support for redistribution	Taxation	High incomes should be taxed more than is currently the case.
McCall et al. (2017)	Support for redistribution	Taxation	The government should increase taxes to give more help to the poor.
McCall et al. (2017)	Support for redistribution	Social spending	We should resist the demands for more benefits from people on welfare. (R)
McCall et al. (2017)	Support for redistribution	Inequality acceptance	In politics, one should strive to assure similar income levels for everyone, regardless of education and employment.
McCall et al. (2017)	Support for redistribution	Government role	The government should intervene economically to redistribute wealth from those who have more resources to those who have fewer resources.
McCall et al. (2017)	Support for redistribution	Social spending	The government spends too much money on the unemployed. (R)
McCall et al. (2017)	Support for redistribution	Redistribution structure	Wealth should be taken from the rich and given to the poor.
McCall et al. (2017)	Support for redistribution	Taxation	Wealthy people should not be taxed more heavily than others. (R)
McCall et al. (2017)	Support for redistribution	Redistribution structure	The wealthy should give more money to those who are worse off.
McCall et al. (2017)	Support for redistribution	Taxation	It is not fair that people have to pay taxes to fund welfare programs. (R)
McCall et al. (2017)	Support for redistribution	Inequality acceptance	Inequality in the distribution of wealth is unjust.
McCall et al. (2017)	Support for redistribution	Redistribution structure	The Israeli fiscal authority should take money from those who have more and give it to those who have less.

Table S1. (*Continued*)

Source	Original concepetulization of the measure	Topic	Items wording
McCall et al. (2017)	Support for redistribution	Taxation	The Israeli government should increase taxes on high earners to give more help to the poor.
McCall et al. (2017)	Support for redistribution	Taxation	Increasing taxes on the wealthy to expand welfare programs can only worsen the current economic situation in Israel. (R)
McCall et al. (2017)	Support for redistribution	Taxation	The well-to-do should not be taxed more heavily than others. (R)
McCall et al. (2017)	Support for redistribution	Redistribution structure	It is very unfair that income is unequally distributed in Israel.
McCall et al. (2017)	Support for redistribution	Government role	The rich should be forced to give more to the poor.
McCall et al. (2017)	Support for redistribution	Social spending	It is not fair that people have to pay taxes to fund welfare programs. (R)
McCall et al. (2017)	Support for redistribution	Taxation	High incomes should be taxed more than is currently the case.
McCall et al. (2017)	Support for redistribution	Social spending	The Israeli government already spends too much money on the unemployed. (R)
vanHeuvelen (2017)	Support for redistribution (ISSP 1)	Government role	"How much do you agree or disagree with each ofthesestatements … It is the responsibility of the government to reduce the differences in income between people with high incomes and those with low incomes"
vanHeuvelen (2017)	Support for redistribution (ISSP 2)	Government role	"On the whole, do you think it should be or should not be the government's responsibility to reduce income differences between the rich and poor" (ISSP 2)
Pratto et al (1998)	Social welfare programs (Sample 3, a = . 68)	Social spending	Govemment-sponsored health care
Pratto et al (1998)	Social welfare programs (Sample 3, a = . 68)	Social spending	Better support for the homeless
Pratto et al (1998)	Social welfare programs (Sample 3, a = . 68)	Social spending	Greater aid to poor children
Pratto et al (1998)	Social welfare programs (Sample 3, a = . 68)	Social spending	Guaranteed jobs for all
Pratto et al (1998)	Social welfare programs (Sample 3, a = . 68)	Social spending	Reduced benefits for the unemployed*
Pratto et al (1998)	Social welfare programs (Sample 3, a = . 68)	Taxation	Increased taxation of the rich
Pratto et al (1998)	Social welfare programs (Sample 3, a = . 68)	Social spending	More support for early education
Pratto et al (1998)	Social welfare programs (Sample 3, a = . 68)	Social spending	Free school lunches
Pratto et al (1998)	Social welfare programs (Sample 3, a = . 68)	Social spending	Low income housing
Pratto et al (1998)	Social welfare programs (Sample 3, a = . 68)	Social spending	Arresting the homeless*

Table S1. (*Continued*)

Source	Original concepetulization of the measure	Topic	Items wording
Lowery (2007)	Support for redistributive policies.	Social policies	In line with previous research (Lowery et al., 2006), attitudes toward redistributive policies were measured by asking participants to rate their support for specific hiring policies. The policies included (a) a preferential recruitment policy, (b) a preferential training policy, (c) a "tiebreaker" policy, (d) a "minimum qualifications" policy, and (e) a "color-blind" policy (reversed). (See Appendix B for the complete text of each policy description.)
			1. A policy whereby a minority group member can get hired over a White applicant as long as the minority group member meets a minimal level of qualifications. Under this policy it is possible for a minority group member to get hired even is he/she is relatively less qualified than a White applicant.
Lowery (2007)	Support for redistributive policies	Social policies	2. A color-blind policy whereby a candidate's race is completely ignored throughout the entire employment procedure (i.e., in both the recruiting and hiring stage).
Lowery (2007)	Support for redistributive policies	Social policies	3. A "tie-breaker" policy in which a minority applicant is selected over a White applicant when the two applicants are equally qualified.
Lowery (2007)	Support for redistributive policies	Social policies	4. A policy through which minority group members can receive supplemental training to prepare them for the selection process. However, minority group status is not considered at the hiring stage
Lowery (2007)	Support for redistributive policies	Social policies	5. A policy that requires an organization to make extra efforts to get members from minority groups to apply for positions but does not take minority status into consideration at the hiring stage. Examples of such extra efforts include advertising in magazines with a high minority readership or recruiting applicants at historically Black colleges and universities.
Shnabel et al. (2016)	support for charity, a non-empowering form of help that was relevant to the Israeli context (α = .85.)	Social policies	"I support civil society organizations whose purpose is providing food for needy Arab families";
Shnabel et al. (2016)	support for charity	Social spending	"I support social initiatives to provide Arab patients with medical equipment such as wheelchairs, breathing machines, etc.";
Shnabel et al. (2016)	support for charity	Social spending	"I'd enjoy hearing the news if it reported the establishment of an organization dedicated to fulfilling wishes forArab children who suffer from cancer";
Shnabel et al. (2016)	support for charity	Social spending	"I support hiring Arab-speaking representatives in the Child Welfare hotline in order to cater for children in need from the Arab sector";

Table S1. (*Continued*)

Source	Original concepetulization of the measure	Topic	Items wording
Shnabel et al. (2016)	support for charity	Social spending	“High priority should be given to ensure that welfare services in the Arab sector are capable ofsheltering women who suffer from domestic abuse”)
Lierse (2018)	perception toward redistribution	Taxation	‘‘Do you think people with high incomes should pay a larger share of their income in taxes than those with low incomes, the same share, or a smaller share?’’
Lierse (2018)	perception toward redistribution	Government role	‘‘to what extent people agree or disagree with whether it is the responsibility of the government to reduce the differences in income between people with high incomes and those with low incomes’’
Lierse (2018)	perception toward redistribution	Taxation	‘‘Generally, how would you describe taxes in <Rs country> today for those with high incomes?’’
Lierse (2018)	perception toward redistribution	Taxation	‘‘Differences in income in <R’s country> are too large’’
Lierse (2018)	perception toward redistribution	Social spending	‘‘if the government should spend less on benefits for the poor’’
Page (2016)	views on redistribution	Inequality acceptance	“Differences in income in America are too large”,
Page (2016)	views on redistribution	Government role	It is the responsibility ofthe government to reduce the differences in income between people with high incomes and those with low incomes”
Newman (2014)	Preferences regarding government redistribution	Government role	‘‘The Government should take measures to reduce differences in income levels.’’
Isakson (2009)	degree of redistribution that the respondents want,	Government role	‘It is the responsibility ofthegovernment to reduce the difference in income betweenpeople with high incomes and those with low incomes’
Kuhn et al (2017)	support for national redistribution	Inequality acceptance	‘Please indicate to what degree you personally agree with the following statements: right now, differences in incomes are too large in [country]
Alesina (2018)	Policy Preferences	Government role	(i) the overall level of government intervention that people would like (through a series of questions presented below)
Alesina (2018)		Social policies	(ii) how a fixed level of revenues should be raised
Alesina (2018)	Policy Preferences<br>We ask respondents to rate their support for the estate tax. Finally, we ask them to allocate 100 percent of the budget to six spending categories:	Social spending	(i) Defense and National Security

Table S1. (*Continued*)

Source	Original concepetulization of the measure	Topic	Items wording
Alesina (2018)	Policy Preferences<br>We ask respondents to rate their support for the estate tax. Finally, we ask them to allocate 100 percent of the budget to six spending categories:	Social spending	(ii) Public Infrastructure
Alesina (2018)	Policy Preferences<br>We ask respondents to rate their support for the estate tax. Finally, we ask them to allocate 100 percent of the budget to six spending categories:	Social spending	(iii) Spending on Schooling and Higher Education
Alesina (2018)	Policy Preferences<br>We ask respondents to rate their support for the estate tax. Finally, we ask them to allocate 100 percent of the budget to six spending categories:	Social spending	(iv) Social Security, Medicare, Disability Insurance, Supplementary Security Income
Alesina (2018)	Policy Preferences<br>We ask respondents to rate their support for the estate tax. Finally, we ask them to allocate 100 percent of the budget to six spending categories:	Social spending	(v) Social Insurance and Income Support Programs
Alesina (2018)	Policy Preferences<br>We ask respondents to rate their support for the estate tax. Finally, we ask them to allocate 100 percent of the budget to six spending categories:	Social spending	(vi) Public Spending on Health
Alesina (2018)	Views on Government	Social policies	"that the government should not concern itself with making the opportunities for children from poor and rich families less unequal," and 7 means "that the government should do everything in its power to reduce this inequality of opportunities."
Alesina (2018)	Views on Government	Taxation	They are also asked whether they think that lowering taxes to stimulate growth or raising taxes to expand programs for the poor would do more to foster equal opportunities
Alesina (2018)	Social spending	Government role	To reduce the inequality of opportunities between children born in poor and rich families, the government has the ability and the tools to do: Nothing at all; Not much; Some; A lot

Table S1. (*Continued*)

Source	Original concepetulization of the measure	Topic	Items wording
Alesina (2018)	Social spending	Social spending	Some people think that the government should not concern itself with making the opportunities for children from poor and rich families less unequal. Others think that the government should do everything in its power to make the opportunities for children from poor and rich families less unequal. Think of a score of 1 as meaning that the government should not concern itself with making the opportunities for children from poor and rich families less unequal, and a score of 7 meaning that the government should do everything in its power to reduce this inequality of opportunities.
Alesina (2018)	Social spending	Social spending	Do you support more policies to increase the opportunities for children born in poor families and to foster more equality of opportunity, such as education policies? Naturally, to finance an expansion of policies promoting equal opportunity, it would have to be the case that either other policies are scaled down or taxes are raised.<br>I very strongly oppose more policies promoting equality of opportunity; I oppose more policies promoting equality of opportunity; I am indifferent; I support more policies promoting equality of opportunity; I very strongly support more policies promoting equality of opportunity.
Alesina (2018)	Social spending	Social spending	In the next two questions, we ask you to think about the total level of funds that the government raises and spends today on various policies. For the purpose of these questions, suppose that the level of government spending is fixed at its current level and cannot be changed. We will ask about your views on two aspects:<br>• First, on the fair split of the tax burden to raise this level of funds.<br>• Second, on how you think the government should spend this level of funds.
Duman (2013)	Support for Government Responsibility	Government role	How would you place your views on this scale? 1 means you agree completely with the statement on the left; 10 means you agree completely with the statement on the right; and if your views fall somewhere in between, you can choose any number in between. The government should take more responsibility to ensure that everyone is provided for versus people should take more responsibility to provide for themselves
Fong (2001)	demand for redistribution	Taxation	People feel differently abut how far a government should go. Here is a phrse which some people believe in adn some don't. Do you think our government should or should not redistribute wealth by heavy taxes on the rich?
Fong (2001)	demand for redistribution	Social spending	Some people feel that the government in Washington, DC should make every possible effort to improve the social and economic position of the poor. Others feel that the government should not make any special effort to help the poor, because they should help themselves. How do you feel about this?

Table S1. (*Continued*)

Source	Original concepetulization of the measure	Topic	Items wording
Fong (2001)	demand for redistribution	Government role	Which one of the following groups do you think has the greatest responsibility for helping the poor: churches, private charities, the government, the families and relatives of poor people, the poor themselves, or someone else?
Fong (2001)	demand for redistribution	Redistribution structure	Do you feel that the distribution ofmoney and wealth in this country today is fair, or do you feel that the money and wealth in this country should be more evenly distributed among a larger percentage of the people?
Fong (2001)	demand for redistribution	Inequality acceptance	Do you think that the fact that some people in the United States are rich and others are poor represents a problem that needs to be fixed or Is an acceptable part of our economic system?
Jaime-Castillo et al (2018)	preference for redistribution	Government role	'the government should take measures to reduce differences in income levels'
Iglesias et al (2013)	This choice of this variable as proxy of demand for redistribution can be justified because liberals and conservatives tend to disagree about the role of luck in financial success, the former thinking it plays a very big role, the latter thinking it plays a small role: that instead financial success is largely attributable to talent and hard work	Ideology	"Being left on the political spectrum".<br>"In political matters, people talk of left and right. How would you place your views on this scale, generally speaking?"
Weakliem (2013)		Taxation	Do you feel there are still a lot of tax loopholes for the rich to avoid taxes, or do you think the rich have to pay higher taxes under our tax system?
Weakliem (2013)		Taxation	If Walter Mondale is elected in November [1984], which federal taxes do you feel likely will be raised – federal taxes on corporations, federal income taxes for everyone, federal income taxes just for the rich, or federal income taxes mainly from middle income people? [multiple responses possible] (Business Week/Harris 1984)
Weakliem (2013)		Taxation	(As you may know, the Federal Tax system has been restructured beginning in 1987. . . .) Who do you think will beneijit most from the (Federal) tax revisions, the rich, the poor, or middle-income people?
Weakliem (2013)		Taxation	Bill Clinton says that under his plan, the rich will pay the largest share of new taxes. Do you think if the Clinton plan is passed, the rich really will pay the most, or do you think they'll ijigure out a way to get around it?

Table S1. (*Continued*)

Source	Original concepetulization of the measure	Topic	Items wording
McCall Kenworthy (2009)	Government responsability toward the poor	Social spending	Should the government do everything possible to improve the standard of living of all poor Americans, or should each person take care of himself?
McCall Kenworthy (2009)	Government responsability toward the poor	Social spending	Should the government reduce income differences between the rich and poor, perhaps by raising taxes of wealthy families or by giving income assistance to the poor, or should the government not concern itself with reducing differences?
McCall Kenworthy (2009)	Government responsability toward the poor	Social spending	We are faced with many problems in this country, none of which can be solved easily or inexpensively. Are we spending too much money, too little money, or about the right amount on welfare?
McCall Kenworthy (2009)	Government responsability toward the poor	Social spending	We are faced with many problems in this country, none of which can be solved easily or inexpensively. Are we spending too much money, too little money, or about the right amount on assistance to the poor?
McCall Kenworthy (2009)	Government responsability to redistribute income	Government role	Do you agree or disagree that it is the responsibility of the government to reduce differences in income between people with high incomes and those with low incomes?
McCall Kenworthy (2009)	Government spending not restricted to the poor	Social spending	We are faced with many problems in this country, none of which can be solved easily or inexpensively. Are we spending too much money, too little money, or about the right amount on improving and protecting the nation's health?
McCall Kenworthy (2009)	Government spending not restricted to the poor	Social spending	We are faced with many problems in this country, none of which can be solved easily or inexpensively. Are we spending too much money, too little money, or about the right amount on health?
McCall Kenworthy (2009)	Government spending not restricted to the poor	Social spending	We are faced with many problems in this country, none of which can be solved easily or inexpensively. Are we spending too much money, too little money, or about the right amount on improving the nation's education system?
McCall Kenworthy (2009)	Government spending not restricted to the poor	Social spending	We are faced with many problems in this country, none of which can be solved easily or inexpensively. Are we spending too much money, too little money, or about the right amount on education?

Table S1. (*Continued*)

Source	Original concepetulization of the measure	Topic	Items wording
McCall Kenworthy (2009)	Government spending not restricted to the poor	Social spending	We are faced with many problems in this country, none of which can be solved easily or inexpensively. Are we spending too much money, too little money, or about the right amount on social security?
McCall Kenworthy (2009)	Taxes	Taxation	Generally, how would describe taxes in American today, meaning all taxes together, including social security, income tax, sales tax, and all the rest: First, for those with high incomes?
Dallinger (2010)	Approval of income redistribution	Inequality acceptance	‘Differences in income in [this country] are too large.’
Dallinger (2010)	Approval of income redistribution	Government role	‘It is the responsibility of the government to reduce the differences in income between people with high income and those with low income.
Gimpelson Treisman (2017)	Preferences over redistribution (ISSP)	Government role	“the responsibility of the government to reduce the differences in income between people with high incomes and those with low incomes.”
Coppini et al (2018)	Beliefs about redistribution	Redistribution structure	Income distribution in Colombia should be more equitable
Coppini et al (2018)	Beliefs about redistribution	Redistribution structure	the government should try to reduce income differences among Colombians.
McCafrey (2006)	Redistribution	Behavioral	Questions differed in whether the way of raising funds was called a "tax" or a "payment," and in whether the distributive properties of the tax/payment were lump sum (same for everyone), progressive (based on ability to pay), or based on use of the service in question.
Linos (2003)	attitudes towards redistribution	Government role	1. ‘It is the responsibility of the government to reduce the di¡erences between people with high incomes and those with low incomes’;
Linos (2003)	attitudes towards redistribution	Social spending	2. ‘The government should provide a job for everyone who wants one’
Linos (2003)	attitudes towards redistribution	Social spending	3. ‘The government should provide everyone with a guaranteed basic income’.

Table S1. (*Continued*)

Source	Original concepetulization of the measure	Topic	Items wording
Boarini (2009)	individual attitudes towards public intervention and to reduce inequality	Government role	(1) Among the following institutions, which one do you think should stand by have-not individuals? • Public Institutions (Central State or LocalAuthorities). • Individuals or Private Foundations/Associations.
Boarini (2009)	individual attitudes towards public intervention and to reduce inequality	Government role	(2) Among the following institutions, which one do you think should mostly battle with poverty? • Public Institutions (Central State or LocalAuthorities). • Individuals or Private Foundations/Associations.
Boarini (2009)	individual attitudes towards public intervention and to reduce inequality	Social spending	(3) Among the following institutions, which one do you think should be made responsible for not self-dependent people? • Public Institutions (Central State or LocalAuthorities). • Individuals or Private Foundations/Associations
Dimick (2018)	Preferences for redistribution	Government role	"The government should take measures to reduce differences in income levels."
McCarthy& Pontusson (2011)	Redistribution (Theoretical)	Social spending	Both measures encompass old-age and disability pensions, unemployment insurance, sick pay and parental leave insurance, family allowances, social assistance, housing subsidies, health care, child care, care for the elderly and disabled, as well as active labor market programs
McCarthy& Pontusson (2011)	Redistribution (Theoretical)	Taxation	The latter measure includes private social expenditures mandated by government as well as the value of tax credits that serve social policy purposes (treating foregone tax revenues as equivalent to government expenditures) and also takes account of direct and indirect taxation of cash benefits received from the government.
Haack & Sieweke (2017)	GGSS to capture the respondents' preferences for redistribution	Social spending	'Should social benefits be cut in the future, should things stay as they are, or should social benefits be extended?'
Haack & Sieweke (2017)	GGSS to capture the respondents' preferences for redistribution	Redistribution structure	'By and large, economic profits are nowadays distributed fairly in Germany'
Choi (2016)	Redistributive preferences	Government role	It is the responsibility of the government to reduce the differences in income between people with high incomes and those with low incomes."
Kim Lee (2018)	attitudes toward redistribution	Government role	"It is the responsibility ofthe government to reduce the differences in income between people with high incomes and those with low incomes."
Kim Lee (2018)	attitudes toward redistribution	Social spending	"The government should provide a decent standard ofliving for the unemployed"
Kim Lee (2018)	attitudes toward redistribution	Social spending	"The government should spend less on benefits forthe poor."

Table S1. (*Continued*)

Source	Original concepetulization of the measure	Topic	Items wording
Scrugs Hayes (2017)	welfare is from the General Social Survey (GSS) cumulative file (1972–2008) while the	Taxation	Unemployment Insurance Replacement Rate (UIRR). We calculate this rate based on the benefit due to a qualified single individual earning the average wage net of federal and state taxes.
Scrugs Hayes (2017)	items for unemployment benefit spending and government spending are from the 2006	Social spending	Listed below are various areas of government spending. Please show whether you would like to see more or less government spending in each area. Remember that if you say “much more”, it might require a tax increase to pay for it. Unemployment Benefits.
Scrugs Hayes (2017)	International Social Survey Programme (ISSP) survey on the role of government.	Social spending	ISSP 2006 Here are some things the government might do for the economy. Please show which actions you are in favor of and which you are against. Government Spending.
Scrugs Hayes (2017)	Welfare ABC News/Washington Post (2010)	Social spending	Because of the economic downturn, Congress has extended the period in which people can receive unemployment benefits, and is considering doing so again. Supporters say this will help those who can’t find work. Opponents say this adds too much to the federal budget deficit. Do you think Congress should or should not approve another extension of unemployment benefits?
Scrugs Hayes (2017)	ABC News/Washington Post (2014)	Social spending	Congress is considering whether or not again to extend the period In which people who’ve lost their jobs can get unemployment benefits. Supporters say this will help those who can’t find work. Opponents say this adds too much to the federal budget deficit. Do you think Congress should or should not approve another extension of unemployment benefits?
Jedinger (2018)	Support for income redistribution	Redistribution structure	“Politics should balance the differences between large and small incomes.”
Jedinger (2018)	Redistribution attitudes.	Government role	“The government should take measures to reduce differences in income levels”
Rosma (2012)	Welfare state dimensions (goal)	Government role	the government should redistribute more to reduce income differences and be responsible for various social security benefits and socials services.
van Oorschot & Meuleman (2012)	Welfare legitimacy: principle of equality	Redistribution structure	‘Large income inequalities are unjust
van Oorschot & Meuleman (2012)	Welfare legitimacy: principle of equality	Government role	Government needs to take substantial measures to reduce income inequalities
van Oorschot & Meuleman (2012)	Welfare legitimacy: principle of activation	Social spending	What should a long-term unemployed do in order to keep his or her benefit?’: Search for a job
van Oorschot & Meuleman (2012)	Welfare legitimacy: principle of activation	Social spending	What should a long-term unemployed do in order to keep his or her benefit?’: Participate in reintegration activities

Table S1. (*Continued*)

Source	Original concepetulization of the measure	Topic	Items wording
van Oorschot & Meuleman (2012)	Welfare legitimacy: principle of activation	Social spending	What should a long-term unemployed do in order to keep his or her benefit?': Get schooling or re-training
van Oorschot & Meuleman (2012)	Welfare legitimacy: principle of activation	Social spending	'What should a young unemployed person do in order to keep his or her benefit?' Search for a job
van Oorschot & Meuleman (2012)	Welfare legitimacy: principle of activation	Social spending	'What should a young unemployed person do in order to keep his or her benefit?' Participate in reintegration activities
van Oorschot & Meuleman (2012)	Welfare legitimacy: principle of activation	Social spending	'What should a young unemployed person do in order to keep his or her benefit?' Take schooling or re-training
van Oorschot & Meuleman (2012)	Welfare legitimacy: role of government- Protection of the weak	Government role	'Government should: reduce income inequalities
van Oorschot & Meuleman (2012)	Welfare legitimacy: role of government- Protection of the weak	Social spending	'Government should: offer more chances for children of poor families to go to university
van Oorschot & Meuleman (2012)	Welfare legitimacy: role of government- Protection of the weak	Social spending	'Government should: spend less on benefits for the poor
van Oorschot & Meuleman (2012)	Welfare legitimacy: role of government- Protection of the weak	Social spending	'Government should: guarantee a reasonable standard of living to unemployed people
van Oorschot & Meuleman (2012)	Welfare legitimacy: role of government- Protection of the weak	Social spending	'Government should: offer a basic minimum income to everybody
van Oorschot & Meuleman (2012)	Welfare legitimacy: role of government - protection against risks	Social spending	Should government organise statutory social benefits to provide for the financial needs that arise for people when being . . . or should it be left to people themselves?' unemployed
van Oorschot & Meuleman (2012)	Welfare legitimacy: role of government - protection against risks	Social spending	Should government organise statutory social benefits to provide for the financial needs that arise for people when being . . . or should it be left to people themselves?' incapacitated for work
van Oorschot & Meuleman (2012)	Welfare legitimacy: role of government - protection against risks	Social spending	Should government organise statutory social benefits to provide for the financial needs that arise for people when being . . . or should it be left to people themselves?' widow(er)
van Oorschot & Meuleman (2012)	Welfare legitimacy: role of government - protection against risks	Social spending	Should government organise statutory social benefits to provide for the financial needs that arise for people when being . . . or should it be left to people themselves?' ill
van Oorschot & Meuleman (2012)	Welfare legitimacy: spending	Social spending	Should government increase or decrease the level of the benefit . . . ? Increase would result in higher contributions, decrease in lower contributions: unemployment benefit

Table S1. (*Continued*)

Source	Original concepetulization of the measure	Topic	Items wording
van Oorschot & Meuleman (2012)	Welfare legitimacy: spending	Social spending	Should government increase or decrease the level of the benefit . . . ? Increase would result in higher contributions, decrease in lower contributions: disability benefit
van Oorschot & Meuleman (2012)	Welfare legitimacy: spending	Social spending	Should government increase or decrease the level of the benefit . . . ? Increase would result in higher contributions, decrease in lower contributions: sick pay
van Oorschot & Meuleman (2012)	Welfare legitimacy: Outcomes	Redistribution structure	How difficult or easy is it for people with benefit . . . to make ends meet?' unemployment benefit
van Oorschot & Meuleman (2012)	Welfare legitimacy: Outcomes	Redistribution structure	How difficult or easy is it for people with benefit . . . to make ends meet?' disability benefit
van Oorschot & Meuleman (2012)	Welfare legitimacy: Outcomes	Redistribution structure	How difficult or easy is it for people with benefit . . . to make ends meet?' sick pay
van Oorschot & Meuleman (2012)	Welfare legitimacy: Outcomes	Redistribution structure	How difficult or easy is it for people with benefit . . . to make ends meet?' old age pension
van Oorschot & Meuleman (2012)	Welfare legitimacy: Outcomes	Redistribution structure	How difficult or easy is it for people with benefit . . . to make ends meet?' minimum benefit
van Oorschot & Meuleman (2012)	Welfare legitimacy: economic and moral consequences	Redistribution structure	Because of the system of social benefits and services . . .' the international competitiveness of the Dutch economy decreases
van Oorschot & Meuleman (2012)	Welfare legitimacy: economic and moral consequences	Redistribution structure	Because of the system of social benefits and services . . .' labour costs increase too much
van Oorschot & Meuleman (2012)	Welfare legitimacy: economic and moral consequences	Redistribution structure	Because of the system of social benefits and services . . .' the economy deteriorates unemployment increases
van Oorschot & Meuleman (2012)	Welfare legitimacy: economic and moral consequences	Redistribution structure	'Because of the system of social benefits and services . . .' people get lazy
van Oorschot & Meuleman (2012)	Welfare legitimacy: economic and moral consequences	Redistribution structure	'Because of the system of social benefits and services . . .' people lose their sense of self-'Because of the system of social benefits and services . . .' responsibility for their subsistence
van Oorschot & Meuleman (2012)	Welfare legitimacy: economic and moral consequences	Redistribution structure	'Because of the system of social benefits and services . . .' people become egoistic and calculative
van Oorschot & Meuleman (2012)	Welfare legitimacy: economic and moral consequences	Redistribution structure	'Because of the system of social benefits and services . . .' people do not want to care for each other anymor

Table S1. (*Continued*)

Source	Original concepetulization of the measure	Topic	Items wording
van Oorschot & Meuleman (2012)	Welfare legitimacy: social consequences	Redistribution structure	‘Because of the system of social benefits and services . . .’ societal unrest is prevented
van Oorschot & Meuleman (2012)	Welfare legitimacy: social consequences	Redistribution structure	‘Because of the system of social benefits and services . . .’ people divorce too easily the
van Oorschot & Meuleman (2012)	Welfare legitimacy: social consequences	Redistribution structure	‘Because of the system of social benefits and services . . .’ Dutch population is happier
van Oorschot & Meuleman (2012)	Welfare legitimacy: social consequences	Redistribution structure	‘Because of the system of social benefits and services . . .’ wealth is distributed more fairly
van Oorschot & Meuleman (2012)	Welfare legitimacy: social consequences	Redistribution structure	‘Because of the system of social benefits and services . . .’ everybody gets a chance to make something of life
Eurobarometer 74.1 Poverty and social exclusion		Government role	Poverty in our country is a problem that needs urgent action by the government
Eurobarometer 74.1 Poverty and social exclusion		Redistribution structure	Nowadays in our country income differences between people are far too large
Eurobarometer 74.1 Poverty and social exclusion		Redistribution structure	The (Nationality) Government should ensure that the wealth of the country is redistributeed in a fair way to all citizens
Eurobarometer 74.1 Poverty and social exclusion		Taxation	People who are well-off should pay higher taxes so the (NATIONALITY) Government has more means to fight poverty
Eurobarometer 74.1 Poverty and social exclusion		Inequality acceptance	There is no point in trying to fitght poverty, it will always exist
Eurobarometer 74.1 Poverty and social exclusion		Inequality acceptance	Income inequalities are necessary for economic developmenet
Eurobarometer 74.1 Poverty and social		Social spending	It is primarily up to the (Nationlity) Government to provide obs for the unemployed

Table S1. (*Continued*)

Source	Original concepetulization of the measure	Topic	Items wording
Eurobarometer 74.1 Poverty and social exclusion		Government role	Providing jobs shuld rest primarily on private companies and mrkets in geneal
Eurobarometer 74.1 Poverty and social exclusion		Social spending	Education should be totally free, even it this means that the quality might be lower
Eurobarometer 74.1 Poverty and social exclusion		Social spending	Tuititio feee are necessary for providing high quality eucation, even if this means that some people won't be able to afford it.
Eurobarometer 74.1 Poverty and social exclusion		Social spending	Higher level of health care, education and social spending must be guaranteed, even if it means that taxes might increase
Eurobarometer 74.1 Poverty and social exclusion		Taxation	Taxes should be decreased even if it means a general lower level of health care, education and social spending
Eurobarometer 74.1 Poverty and social exclusion		Government role	The (Nationlity) Government should take more resopnsability to ensure that everyone is provided for
Eurobarometer 74.1 Poverty and social exclusion		Government role	People shuld take more responsability to provide for themselves
Eurobarometer 74.1 Poverty and social exclusion		Redistribution structure	The (NATIONALITY) Government should ensure that the wealth of the country is redistributed in a fair way to all citizens
Eurobarometer 74.1 Poverty and social exclusion		Taxation	People who are well-off should pay higher taxes so the (NATIONALITY)
Eurobarometer 74.1 Poverty and social exclusion		Government role	Government has more means to fight poverty

Table S1. (*Continued*)

Source	Original concepetulization of the measure	Topic	Items wording
Eurobarometer 81.5 Social climate		Social spending	It is primarily up to the (Nationlity) Government to provide obs for the unemployedd
Eurobarometer 81.5 Social climate		Government role	It is primarily up to private companies and markets in general to provide jobs fot the unemployed
Eurobarometer 81.5 Social climate		Social spending	Education should be totally free, even if this means that the quality might be lower
Eurobarometer 81.5 Social climate		Social spending	Tuition fees are necessary for providing high quality education, even if this means that some people won't be able to afford it
Eurobarometer 81.5 Social climate		Social spending	A high level of health care, education and social spending must be guaranteed, even if it means that taxes might increase
Eurobarometer 81.5 Social climate		Taxation	Taxes should be decreased even if it means a general lower level of heealth care, education and social spending
Eurobarometer 81.5 Social climate		Government role	The (Nationlity) Government should take more resopnsability to ensure that everyone is provided for
Eurobarometer 81.5 Social climate		Government role	People shuld take more responsability to provide for themselves
Eurobarometer 72.1 Poverty social exclusion and economy		Inequality acceptance	Poverty in our country is a problem that needs urgent action by the government
Eurobarometer 72.1 Poverty social exclusion and economy		Inequality acceptance	Nowadays in our country income differences between people are far too large
Eurobarometer 72.1 Poverty social exclusion and economy		Redistribution structure	The (Nationality) Government should ensure that the wealth of the country is redistributeed in a fair way

Table S1. (*Continued*)

Source	Original concepetulization of the measure	Topic	Items wording
Eurobarometer 72.1 Poverty social exclusion and economy		Taxation	People who are well off shouldpay higher taxes so the Nationality Government has more menas to fight poverty
Eurobarometer 72.1 Poverty social exclusion and economy		Inequality acceptance	There is no point in trying to fight poverty, it will always exist
Eurobarometer 72.1 Poverty social exclusion and economy		Social spending	It is primarily up to the (Nationlity) Government to provide obs for the unemployedd
Eurobarometer 72.1 Poverty social exclusion and economy		Government role	It is primarily up to private companies and markets in general to provide jobs fot the unemployed
Eurobarometer 72.1 Poverty social exclusion and economy		Social spending	Education should be totally free, even if this means that the quality might be lower
Eurobarometer 72.1 Poverty social exclusion and economy		Social spending	Tuition fees are necessary for providing high quality education, even if this means that some people won't be able to afford it
Eurobarometer 72.1 Poverty social exclusion and economy		Social spending	A high level of health care, education and social spending must be guaranteed, even if it means that taxes might increase
Eurobarometer 72.1 Poverty social exclusion and economy		Taxation	Taxes should be decreased even if it means a general lower level of heealth care, education and social spending

Table S1. (*Continued*)

Source	Original concepetulization of the measure	Topic	Items wording
Eurobarometer 72.1 Poverty social exclusion and economy		Government role	The (Nationlity) Government should take more resopnsability to ensure that everyone is provided for
Eurobarometer 72.1 Poverty social exclusion and economy		Government role	People shuld take more responsability to provide for themselves
General Social Survey 2015	Support for policies aimed to reduce inequality	Taxation	Question: Some people think that the government in Washington ought to reduce the income differences between the rich and the poor, perhaps by raising the taxes of wealthy families or by giving income assistance to the poor. Others think that the government should not concern itself with reducing this income difference between the rich and the poor. Here is a card with a scale from 1 to 7. Think of a score of 1 as meaning that the government ought to reduce the income differences between rich and poor, and a score of 7 meaning that the government should not concern itself with reducing income differences. What score between 1 and 7 comes closest to the way you feel?
General Social Survey 2015	Support for policies aimed to reduce inequality	Social spending	Question: Some people think that the government in Washington should do everything possible to improve the standard of living of all poor Americans; they are at Point 1 on this card. Other people think it is not the government's responsibility, and that each person should take care of himself; they are at Point 5. Where would you place yourself on this scale, or haven't you made up your mind on this?
General Social Survey 2015	Support for policies aimed to reduce inequality	Social spending	Question: We are faced with many problems in this country, none of which can be solved easily or inexpensively. I'm going to name some of these problems, and for each one I'd like you to tell me whether you think we're spending too much money on it, too little money, or about the right amount. Are we spending too much, too little, or about the right amount on [Welfare/Assistance to the Poor]?

Table S1. (*Continued*)

Source	Original concepetulization of the measure	Topic	Items wording
Euroean Social Survey Wave7 2014		Government role	The government should take measures to reduce differences iin income levels
ISSP 2009 Social inequality module		Government role	It is the responsability of the government to reduce the differences in income between eople with high incomes and those with low incomes
ISSP 2009 Social inequality module		Social spending	The government should provide a decent standard of livingfor the unemployed
ISSP 2009 Social inequality module		Social spending	The government should sepend less on benefits for the poor.
ISSP 2009 Social inequality module		Taxation	Do you think people with high incomes should pay a largeer share of their income in taxes than those with low incomes, the same share, or a smaller share
ISSP 2009 Social inequality module		Taxation	Generally, how would you describe taxes in <countru> today for those with high incomes? Taxes are much too high, too high, about right, too low, much too low.
ISSP 2009 Social inequality module		Social spending	Is is just or unjust -right or wrong- that people with hihger incomes can buy better health care than peoople with lower incomes? Very just, definitely right to very unjust, definitely wrong
ISSP 2009 Social inequality module		Social spending	Is is just or unjust -right or wrong- that people with hihger incomes can buy better education for their children than peoople with lower incomes? Very just, definitely right to very unjust, definitely wrong
ISJP		Social spending	The government should guarantee everyone a minimum standard of living.
ISJP		Redistribution structure	The fairest way of distributing wealth and income would be to give everyone equal shares.
ISJP		Government role	The government should place an upper limit on the amount of money any one person can make.
ISJP		Social spending	The government should provide a job for everyone who wants one.
ISJP		Social spending	It is just that disadvantaged groups are given extra help so that they can have equal opportunities in life.
ISJP		Redistribution structure	The most important thing is that people get what they need, even if this means allocating money from those who have earned more than they need

Table S1. (*Continued*)

Source	Original concepetulization of the measure	Topic	Items wording
Latinobarómetro 2013		Government role	Con una escala de 1 a 10, donde 1 es "cada cual se debe hacer cargo de su propio bienestar" y 10 "es el gobierno el que debe asumir la responsabilidad del bienestar de la gente" ¿Dónde se ubica Ud.? CADA CUAL SE DEBE HACER CARGO DE SU PROPIO BIENESTAR EL GOBIERNO DEBE ASUMIR LA RESPONSABILIDAD DEL BIENESTAR DE LA GENTE
Pew Research Center 2014 Global Attitudes Questionnaire		Taxation	What would do more to reduce the gap between the rich and the poor in our country? High taxes on the wealthy and corporations to fund programs that help the poor OR Low taxes on the wealthy and corporations to encourage investment and economic growth?<br>1 High taxes to fund programs for poor<br>2 Low taxes to encourage investment and growth
WVS6 2012		Government role	VGovernment should take more responsability to ensure that everyone is provided for OR People should tke more responsability to provide for themselves
WVS6 2012		Government role	Private ownership of business and industry should be increased OR Government ownership of busiiness and industry should be increased
NZAVS		Taxation	Redistributing money and wealth more evenly among a larger percentage of the people in New Zealand through heavy taxes on the rich.
LAPOP merge data 2004-2014		Government role	The [country] government should implement strong policies to reduce income inequality between the rich and the poor. To what extent do you agree or disagree
LAPOP merge data 2004-2014		Taxation	You would be willing to pay more taxes if they were used to give more to those who have less. To what extent do you agree or disagree?
COES - ENACOES		Redistribution structure	Las grandes empress deberían hacer algo para reducir las diferencisa salariales entre los trabajadores de altos salarios de aquellos con bajos salarios
COES - ENACOES		Taxation	¿Cree usted que las personas con altos ingresos deberían pagar en impuestos una proporción mucho menor, menor, igual, mayor o mucho mayor que aquellas personas con bajos ingresos?
COES - ENACOES		Taxation	En general, ¿Cómo describiría usted los impuestos en Chile actual para aquellos con altos ingresos? Muy Bajos hasta Muy altos

Table S1. (*Continued*)

Source	Original concepetulization of the measure	Topic	Items wording
COES - ENACOES		Social spending	Es just que las personas con mayores ingresos puedan tener mejores pensioens que otras personas
COES - ENACOES		Social spending	Es justo que aquellos que pueden pagar tengan una buena educacion para sus hijos
COES - ENACOES		Government role	El gobierno debería hacer algo para reducir las diferencias de ingreso entre ricos y pobres
COES - ENACOES		Social spending	Es justo que las personas con mayores ingresos puedan acceder a una mejor atención de salud que las personas con ingresos más bajos

Table S2. Descriptive statistics for the items of support for redistribution

No.	Item (spanish)	Item (english)	N	M	SD	Med.	Range	Skew
1	El Gobierno tiene la responsabilidad de reducir las diferencias de ingresos entre los que tienen más y los que tienen menos.	The government has a responsibility to reduce the income gap between those who have more and those who have less.	1932	5.33	1.55	6	6 (1-7)	-0.90
3	El Gobierno debería gastar más dinero en subsidios para los pobres.	The government should spend more money on subsidies for the poor.	1932	4.16	1.83	4	6 (1-7)	-0.15
4	El Gobierno debería imponer mayores impuestos a las personas con más ingresos económicos.	The government should impose higher taxes on people with the highest income.	1929	5.15	1.74	6	6 (1-7)	-0.81
5	Se deberían reservar cupos en universidades para las personas más desfavorecidas.	Places in universities should be reserved for the most disadvantaged people.	1929	5.73	1.49	6	6 (1-7)	-1.29
6	Existe una gran necesidad de redistribuir la riqueza de aquellos que tienen más, hacia aquellos que tienen menos.	There is a great need to redistribute wealth from those who have more to those who have less.	1932	4.88	1.71	5	6 (1-7)	-0.61
7	No hay ninguna necesidad de cambiar la distribución de ingresos económicos en Colombia.	There is no need to change the distribution of economic income in Colombia.	1928	2.03	1.50	1	6 (1-7)	1.60
8	El gobierno debería incrementar los impuestos para dar más ayudas a las personas más necesitadas.	The government should increase taxes to give more aid to those most in need.	1933	3.32	1.82	3	6 (1-7)	0.34
9	Las personas con más riqueza deberían ayudar más a las personas más necesitadas.	The richest people should help the most needy people more.	1928	5.36	1.48	6	6 (1-7)	-0.79

Table S3. Descriptive statistics for the items of support for redistribution and inter-item correlation in Study 2

	Descriptive and reliability				Inter-item correlation					
Item	Mean	SD	Skew	α if deleted	*redis1*	*redis3*	*redis4*	*redis5*	*redis6*	*redis7r*
redis1	5.33	1.55	-0.9	0.643						
redis3	4.16	1.83	-0.15	0.703	0.178***					
redis4	5.15	1.74	-0.81	0.658	0.344***	0.124***				
redis5	5.73	1.49	-1.29	0.672	0.241***	0.220***	0.238***			
redis6	4.88	1.71	-0.61	0.624	0.452***	0.204***	0.395***	0.247***		
redis7r	5.97	1.5	-1.6	0.703	0.256***	0.017	0.160***	0.129***	0.266***	
redis9	5.36	1.48	-0.79	0.641	0.306***	0.326***	0.326***	0.329***	0.390***	0.126***

Table S4. Correlations between latent variables of support for redistribution and other variables

	Support for redistribution (general)	Dimensions of support for redistribution	
	General scale (8 items)	Support for redistribution (taxing the wealthy)	Support for redistribution (assisting the poor)
Support for redistribution (assisting the poor)	-	-	0.75***
Perceived income gap	0.17***	0.20***	0.06
Ideal income gap	-0.17***	-0.17***	-0.10**
Perceived inequality in daily-life	0.10***	0.07*	0.14***
General concern of inequality	0.16***	0.14***	0.16***
Meritocracy	0.03	-0.01	0.10**
Economic system justification	-0.05	-0.07*	-0.00
Political ideology (left-right)	-0.22***	-0.26***	-0.08*
SES by income	-0.14***	-0.15***	-0.07*
SSS	-0.10***	-0.11***	-0.05
SES by education	0.00	0.02	-0.04
Sex	-0.04	-0.04	-0.02
Age	0.07*	0.08**	0.04

Note: *** p <. 001; ** p <.01, *p <. .05
